# Supplementary material for: Patterns of aDNA damage through time and environments—lessons from herbarium specimens
Source: Gigascience. 2026 Mar 5;15:giag026. doi: 10.1093/gigascience/giag026 (PMC13108254; doi:10.1093/gigascience/giag026)
Supplement: giag026_GIGA-D-25-00447_original_submission [file giag026_giga-d-25-00447_original_submission.pdf]

GigaScience

Patterns of aDNA Damage Through Time end Environments – lessons from herbarium specimens

--Manuscript Draft--

**Manuscript Number:**	GIGA-D-25-00447
**Full Title:**	Patterns of aDNA Damage Through Time end Environments – lessons from herbarium specimens
**Article Type:**	Research
**Funding Information:**	European Union Horizon 2020 research and innovation programme (862613) — Dr Nils Stein<br>UK Research and Innovation (EP/X022404/1) — Dr Rafal M. Gutaker<br>King Abdullah University of Science and Technology (ORA-CRG10-2021-4734) — Dr Rod A. Wing
**Abstract:**	Herbarium collections are a vast but underutilized resource for ancient DNA research, containing over 400 million specimens with detailed metadata and spanning centuries of global biodiversity. Understanding patterns of DNA preservation in natural collections is crucial for optimizing ancient DNA studies and informing future curation practices. We analysed genomic data for 573 herbarium specimens from six plant species from the genera Hordeum and Oryza collected from the Americas and Eurasia over 220 years using standardized laboratory protocols and shotgun sequencing to quantify DNA degradation and elucidate factors that accelerate it. We find significant age-dependent DNA fragmentation rates, indicating temporal degradation processes not detected in prehistoric samples. In our analysis, DNA decay rates in herbarium specimens were almost eight times faster than in moa bones, reflecting fundamental differences in tissue composition and preservation environments. Environmental conditions at the time of specimen collection emerged as the major determinants of post-mortem mutation rates, with temperature being the dominant driver of cytosine deamination. We find no effect of sample storage on DNA degradation. These findings provide insights into how climatic origin, preservation environment, taxonomic identity and age influence DNA preservation while highlighting opportunities for improving institutional preservation practices. Due to standardized preservation conditions, museum collections can provide better insights into DNA degradation over time than archaeological and paleontological samples.
**Corresponding Author:**	Stefano Porrelli<br>Royal Botanic Gardens Kew<br>London, UNITED KINGDOM
**Corresponding Author Secondary Information:**	
**Corresponding Author's Institution:**	Royal Botanic Gardens Kew
**Corresponding Author's Secondary Institution:**	
**First Author:**	Stefano Porrelli
**First Author Secondary Information:**	
**Order of Authors:**	Stefano Porrelli
	Alice Fornasiero
	Phuong Hong Le
	Whenzhe Yin
	Maria Navarrete Rodriguez
	Nahed Mohammed

Powered by Editorial Manager® and ProduXion Manager® from Aries Systems Corporation

	Axel Himmelbach
	Andrew C. Clarke
	Nils Stein
	Paul J. Kersey
	Rod A. Wing
	Rafal M. Gutaker
**Order of Authors Secondary Information:**	
**Additional Information:**	
**Question**	**Response**
Are you submitting this manuscript to a special series or article collection?	No
**Experimental design and statistics**<br><br>Full details of the experimental design and statistical methods used should be given in the Methods section, as detailed in our Minimum Standards Reporting Checklist. Information essential to interpreting the data presented should be made available in the figure legends.<br><br>Have you included all the information requested in your manuscript?	Yes
**Resources**<br><br>A description of all resources used, including antibodies, cell lines, animals and software tools, with enough information to allow them to be uniquely identified, should be included in the Methods section. Authors are strongly encouraged to cite Research Resource Identifiers (RRIDs) for antibodies, model organisms and tools, where possible.<br><br>Have you included the information requested as detailed in our Minimum Standards Reporting Checklist?	Yes
**Availability of data and materials**<br><br>All datasets and code on which the	Yes

conclusions of the paper rely must be either included in your submission or deposited in publicly available repositories (where available and ethically appropriate), referencing such data using a unique identifier in the references and in the “Availability of Data and Materials” section of your manuscript.<br><br>Have you have met the above requirement as detailed in our Minimum Standards Reporting Checklist?	
GigaScience has policies and guidelines in place for the use of generative AI-writing tools such as ChatGPT. If you have used such writing tools to assist with writing the manuscript this must be declared and cited in the text. Authors should not list AI-writing tools and other AI-assisted technologies as an author or co-author and should acknowledge that they are fully responsible for text generated or refined by AI-writing tools.<p><br><br>A summary of use (particularly in the introduction or among methods) needs to be included at the end of the paper, and the outputs should also be included as a supplementary file hosted in GigaDB or other open repositories. Please read our guidelines for more information. <p><br><br>By submitting to GigaScience, you are aware of the journal's AI-writing tools policy, and if you have declared use of such tools below, you have acknowledged this where appropriate in your manuscript and have made a summary of use and outputs available. <p><br><b>AI-assisted writing tools have been used in the preparation of this manuscript?	No

Manuscript

Click here to access/download;Manuscript;aDNA_Damage_v6.docx

Manuscript title:

Patterns of aDNA Damage Through Time end Environments – lessons from herbarium specimens

Authors:

Stefano Porrelli[1,*], ORCID: 0000-0003-3878-7745

Alice Fornasiero[2], ORCID: 0000-0001-6165-4233

Phuong Hong Le[1], ORCID: 0000-0002-2862-6502

Whenzhe Yin[3,4], ORCID: 0000-0002-4350-3578

Maria Navarrete Rodriguez[2], ORCID: 0000-0001-5052-3713

Nahed Mohammed[2], ORCID: 0000-0002-8857-3246

Axel Himmelbach[5], ORCID: 0000-0001-7338-0946

Andrew C. Clarke[3], ORCID: 0000-0003-2293-1423

Nils Stein[5,6], ORCID: 0000-0003-3011-8731

Paul J. Kersey[1], ORCID: 0000-0002-7054-800X

Rod A. Wing[2,7] ORCID: 0000-0001-6633-6226

Rafal M. Gutaker[1,*], ORCID: 0000-0001-9226-879X

[1] Trait Diversity and Function, Royal Botanic Gardens, Kew, Richmond, TW9 3AE, United Kingdom.

[2] Plant Science Program, Biological and Environmental Science and Engineering Division, King Abdullah University of Science and Technology (KAUST), Thuwal, Saudi Arabia.

[3] School of Biosciences, University of Nottingham, Sutton Bonington, LE12 5RD, United Kingdom.

[4] State Key Laboratory of Emerging Infectious Diseases, School of Public Health, The University of Hong Kong, Hong Kong, SAR, China.

[5] Genebank, Leibniz Institute of Plant Genetics and Crop Plant Research (IPK), Seeland, 06466, Germany.

[6] Institute of Agricultural and Nutritional Sciences, Martin Luther University of Halle-Wittenberg, Halle (Saale), Germany.

[7] Arizona Genomics Institute, School of Plant Sciences, University of Arizona, Tucson, AZ, USA.

* To whom correspondence should be addressed: R.Gutaker@kew.org; S.Porrelli2@kew.org

Abstract:

Herbarium collections are a vast but underutilized resource for ancient DNA research, containing over 400 million specimens with detailed metadata and spanning centuries of global biodiversity. Understanding patterns of DNA preservation in natural collections is crucial for optimizing ancient DNA studies and informing future curation practices. We analysed genomic data for 573 herbarium specimens from six plant species from the genera *Hordeum* and *Oryza* collected from the Americas and Eurasia over 220 years using standardized laboratory protocols and shotgun sequencing to quantify DNA degradation and elucidate factors that accelerate it. We find significant age-dependent DNA fragmentation rates, indicating temporal degradation processes not detected in prehistoric samples. In our analysis, DNA decay rates in herbarium specimens were almost eight times faster than in moa bones, reflecting fundamental differences in tissue composition and preservation environments. Environmental conditions at the time of specimen collection emerged as the major determinants of post-mortem mutation rates, with temperature being the dominant driver of cytosine deamination. We find no effect

of sample storage on DNA degradation. These findings provide insights into how climatic origin, preservation environment, taxonomic identity and age influence DNA preservation while highlighting opportunities for improving institutional preservation practices. Due to standardized preservation conditions, museum collections can provide better insights into DNA degradation over time than archaeological and paleontological samples.

Introduction:

Understanding preservation of DNA from old biological samples, commonly referred to as ancient DNA (aDNA), has been at the core of the genomic revolution in archaeological research [1,2]. There is a consensus among researchers that aDNA is defined by its degradation and not by its age [3,4], though the two are correlated [5]. The two most prominent reactions associated with DNA degradation are deamination (resulting with spontaneous substitutions of cytosine residues to uracil) and depurination (breakage of the phosphodiester bond resulting in DNA backbone fragmentation). Both reactions occur spontaneously in DNA in the absence of enzymatic repair machinery [6,7]. Deamination leads to a characteristic pattern of ‘C>T’ substitutions in DNA fragments, with increased frequency at fragment termini, while depurination leads to DNA fragmentation towards very small molecules (~30-100 bp). Both patterns are common in historical, archaeological and sedimentary samples [2,5,8] and make bioinformatic processing and downstream analyses challenging [9]. Although C>T misincorporations can be used to authenticate genuine aDNA sequences, they can bias variant calling and phylogenetic analyses, potentially leading to incorrect inferences [3]. Similarly, extensive DNA fragmentation reduces mapping efficiency and increases the likelihood of spurious alignments [10,11]. These challenges are particularly acute for ancient samples from hot and humid environments, where accelerated degradation processes further compromise DNA integrity [12,13]. As a result, tools and approaches have been developed to improve

quality control, reads processing, mapping and downstream analyses in a quest to utilize even highly degraded samples [14–16]. In addition to chemical degradation, DNA can also be altered through biological processes such as microbial colonization. In effect, the *bona fide* DNA from the target species can be depleted and replaced with post-mortem microbial DNA [17].

While the vast majority of published aDNA research focuses on human skeletal remains [18,19], there are an increasing number of studies in other mammals [20], arthropods [8,21], and in plants [22,23]. Archaeobotanical materials, primarily seeds, are often used as sources of prehistoric DNA. While not older than 500 years, herbarium specimens number over 400 million [24], and have solid species identification and, frequently, collection metadata. They comprise a vast but underutilised resource for studying evolutionary and ecological changes in the last couple of centuries [25,26]. The leaf tissue is most often the target in DNA isolation protocols but does not possess the same structural isolation from the environment as bones or seeds. This is the most likely reason why DNA in herbarium samples decays at a rate roughly six times faster than in ancient Moa bones [27], but also more than twice as slow when compared to dry-pinned arthropod museum specimens [8]. On the other hand, herbarium specimens are commonly dried upon collection and are generally stored in standardized conditions that are favourable for DNA preservation, such as stable temperatures and low air humidity. Availability of materials, good geolocation information and relatively stable preservation condition ex situ make herbarium specimens a perfect system to study the effects of the environment at the point of collection on the subsequent preservation of DNA in biological samples.

One of the major challenges in studies trying to investigate the dynamics of DNA mutation, decay and fragmentation is lack of large datasets with a global distribution and consistent sampling and laboratory processing procedures. Previous studies were limited to one or two

species representing narrow geographical range [12,27]. Here, we present new sequencing data for a total of 573 herbarium samples from 6 plant species, spanning the Americas and Eurasia, processed with the same laboratory protocol and sequenced using a whole-genome shotgun approach. Our main aim is to understand which environmental factors influence the rate of DNA mutation and decay. The outcome of this investigation brings new insights into the fundamental processes of DNA degradation that can inform future conservation of museum specimens, as well as help researchers to prioritize materials for their studies.

Methods

Herbarium samples

Historical herbarium samples (N = 573) were obtained from seven herbaria in Europe and North America (table 1): Royal Botanic Gardens, Kew (UK), Smithsonian Institute Herbarium (US), Missouri Botanic Gardens (US), New York Botanic Gardens (US), National History Museum (France), Harvard University Herbarium (US), and National History Museum (UK). Passport information for each sample is included in the supplementary material (supplementary table S1). The *Hordeum* samples were predominantly collected from temperate Europe to semi-arid regions of the Middle East, with highest counts from countries such as Iraq, Israel and Iran. The *Oryza* samples were predominantly collected from tropical and subtropical regions of the Americas and Southeast Asia, particularly Brazil, Costa Rica, Colombia, Mexico and Thailand (figure 1).

Table 1: Number of historical herbarium samples (N = 573) included in this study collected from seven herbaria. RBGK: Royal Botanic Gardens Kew, UK; SIHUS: Smithsonian Institute Herbarium, US; MBG: Missouri Botanic Gardens, US; NYBGUS: New York Botanic Gardens,

US; NHMFR: National History Museum, France; HUHUS: Harvard University Herbarium, US; NHMUK: National History Museum, UK.

Species	RBGK	SIHUS	MBG	NYBGUS	NHMFR	HUHUS	NHMUK	Total	Age range
Hordeum spontaneum	146	0	0	0	0	0	0	146	1822-2017
Hordeum vulgare	82	0	0	0	0	0	0	82	1819-2015
Oryza alta	0	2	9	0	2	0	0	13	1913-2002
Oryza grandiglumis	1	30	1	0	6	0	0	38	1930-2002
Oryza latifolia	22	97	36	30	20	16	10	231	1797-2011
Oryza rufipogon	29	31	3	0	0	0	0	63	1909-1998
**Total**	280	160	49	30	28	16	10	573	1797-2017

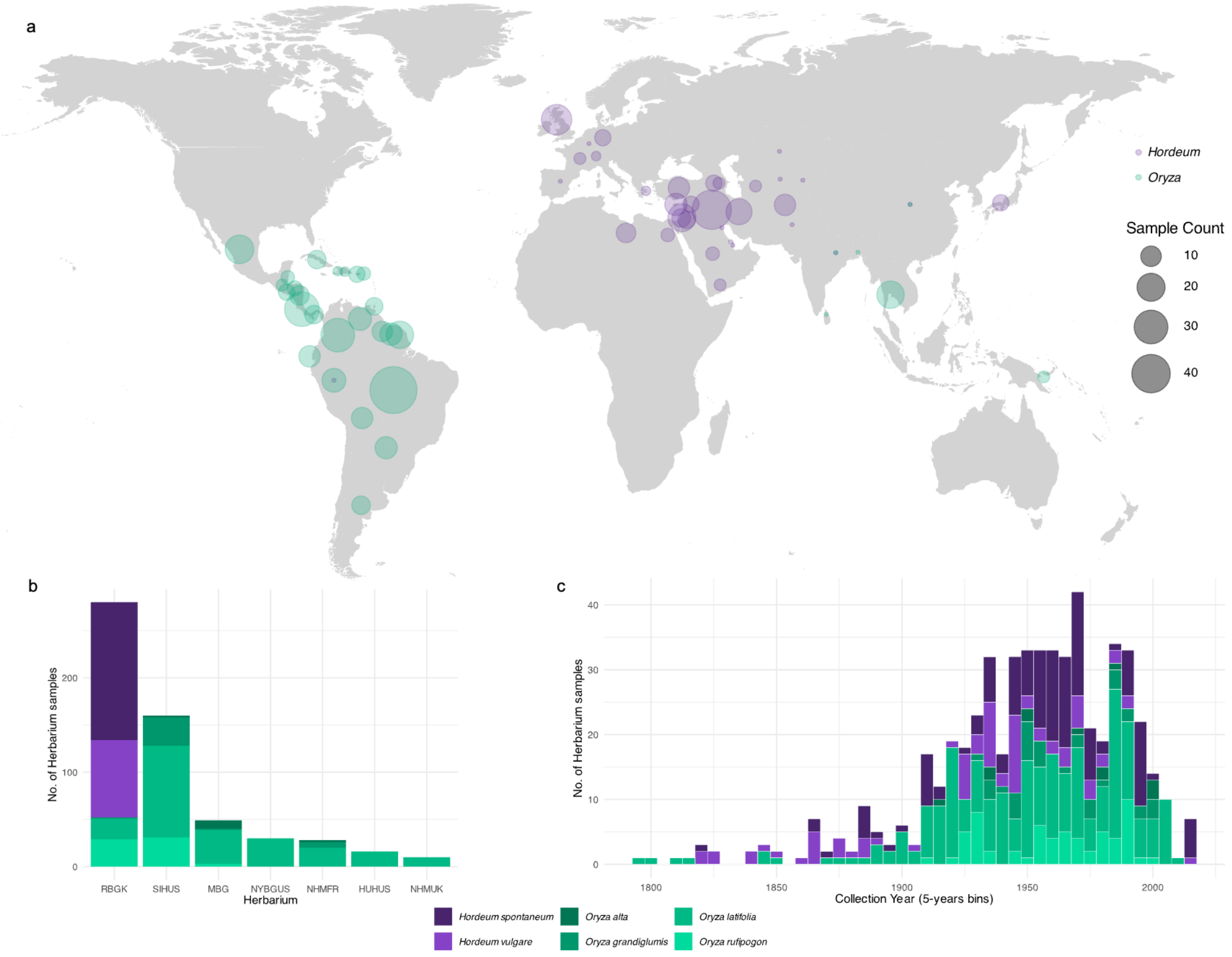

Figure 1: Overview of the herbarium samples of barley (*Hordeum*) and rice (*Oryza*) species: (a) geographical distribution based on country of collection; (b) source of herbarium of *Hordeum* and *Oryza* species (RBGK: Royal Botanic Gardens Kew, UK; SIHUS: Smithsonian Institute Herbarium, US; MBG: Missouri Botanic Gardens, US; NYBGUS: New York Botanic Gardens, US; NHMFR: National History Museum, France; HUHUS: Harvard University Herbarium, US; NHMUK: National History Museum, UK); (c) temporal distribution for each species.

DNA extraction, library preparation and low throughput sequencing:

Laboratory steps for DNA extraction and library preparation for all 228 *Hordeum* (146 *H. spontaneum* and 82 *H. vulgare)* and 58 Oryza (30 *O. latifolia,* 18 *O. rufipogon* and 10 *O. grandiglumis*) (Table 1) samples were carried out in clean room facilities at Royal Botanic Gardens, Kew (UK) following previously published best practices and aDNA protocol described in [28] with adjusted volumes. Tissue samples (5-10 mg) were grinded in 2mL PowerBead metal tubes (QIAGEN, 13117-50) with a bead mill homogeniser (Precellys Evolution, P002511-PEVT0-A.0). 1mL of PTB-based mix was added to the homogenized samples, which were then incubated on a rotor overnight at 37°C. gDNA was isolated and half the volume (0.5 mL) was purified using the DNeasy Plant Mini Kit (QIAGEN, 69106) with modifications described in [28], and quantified with Quantus™ fluorometer (Promega, E6150). For a subset of the gDNA isolates (*N*=40, 10 *H. spontaneum*, 10 *H. vulgare*, 10 *O. rufipogon*, and 10 *O. latifolia*; supplementary table S2), the genomic DNA fragment size distribution was analysed with the 4200 TapeStation system (Agilent, G2991BA) using a D1000 ScreenTape (Agilent, 5067-5582).

Double stranded and doubled indexed genomic libraries were constructed by blunt-end DNA ligation [29,30] following [28]. Briefly, genomic DNA was blunt-end repaired, and universal Illumina double-stranded adapters were ligated. This was followed by fill-in of adapters, indexing with unique combinatorial Illumina indexes, and amplification. Between each step, samples were purified with the MiniElute® PCR purification kit (QIAGEN, 28006). To quantify the excess of C>T substitutions at reads termini caused by spontaneous deamination of cytosines [31], and to validate aDNA authenticity [27,32], we did not perform enzymatic removal of aDNA-associated DNA misincorporation [33]. Indexed and amplified libraries were quantified with Quantus™ fluorometer (Promega, E6150). fragment size of each library were estimated with a 4200 TapeStation System (Agilent, G2991BA) using a D1000 ScreenTape (Agilent, 5067-5582). Libraries were pooled equimolarly and sequenced in paired-end mode. All *Hordeum* libraries were sequenced on a NovaSeq 6000 or NovaSeq X+ device (2 x 62 bp) according to manufacturer's instructions (Illumina, San Diego, CA, USA) at the Leibniz Institute of Plant Genetics and Crop Plant Research (IPK, Gatersleben, Germany). 40 *Oryza* libraries were sequenced at Macrogen (Macrogen, Europe), on an Illumina Novaseq X+ system (2 x 150 bp). A further 287 *Oryza* samples were obtained with fundings from the King Abdullah University of Science and Technology (KAUST). These samples were processed in a clean room facility at the Ancient and Environmental DNA Laboratory (ÆDNA) at the University of Nottingham (UoN, UK) following the same protocol, and sequenced on an Illumina Miseq platform (2 x 150 bp) according to manufacturer's instructions (Illumina, San Diego, CA, USA) at the Deep Seq facility at UoN.

Bioinformatic screening and validation of aDNA authenticity

Bioinformatics protocols for read processing, quality assessment, screening and authentication of aDNA-derived libraries follow [28]. Demultiplexed raw Illumina paired-end reads were

adapter trimmed and merged with AdapterRemoval v2.3.4 [34] with a minimum overlap of 11 bases, and quality-checked with FastQC v0.11.8 (http://www.bioinformatics.bbsrc.ac.uk/projects/fastqc). Reference genome assemblies were retrieved from the National Center for Biotechnology Information (NCBI) database (table 2) and indexed with BWA v0.7.19 [35] and SAMtools v1.21 [36]. Trimmed and merged reads were mapped to their respective reference genome with BWA-aln using aDNA-specific parameters ("-l 1024"). PCR optical duplicate reads removed with DeDup [16]. Base frequencies at DNA break points and nucleotide misincorporations were estimated for each library using MapDamage2 v2.0.6 [37]. Mapping statistics of the additional 287 *Oryza* samples were obtained by mapping sequencing reads to the following reference genomes: *O. alta* (PRJNA1039467), *O. grandiglumis* (PRJNA737282), *O. latifolia* (PRJNA737486) [38]. The *O. rufipogon* samples originally collected in the American continent were aligned to the *O. glumipatula* genome reference (PRJNA48429).

Table 2: Reference genome assemblies used in this study with associated BioProject, BioSample and Accession IDs.

**Reference**	**BioProject**	**BioSample**	**Assembly Accession**
Hordeum spontaneum	PRJEB57567	SAMEA112465237	GCA_949783245.1
Hordeum vulgare	PRJEB40589	SAMEA7384724	GCF_904849725.1
Oryza alta	PRJNA1039467	SAMN38217704	GCA_047899615.1
Oryza grandiglumis	PRJNA737282	SAMN19687255	GCA_048188845.1
Oryza latifolia	PRJNA737486	SAMN19696891	GCA_048174585.1
Oryza rufipogon	PRJNA1029807	SAMN40302812	GCA_037997075.1
Oryza glumipatula	PRJNA48429	SAMN02981440	GCA_000576495.2

Ancient DNA damage metrics and regression analyses

Four metrics were selected to quantify patterns of aDNA damage: (i) the proportion of endogenous DNA content, (ii) the fragment length distribution, (iii) the damage fraction per site (λ), and (iv) the frequencies of 5' C>T substitutions. The four metrics were analysed in linear models as a function of collection year and sample age using the '*lm*' function in R [39]. The DNA decay rates (*k*) of *Hordeum* and *Oryza* samples were also determined independently.

Endogenous fraction

The percentage of post-quality trimmed and merged reads for each sample mapped to its respective reference was used as a proxy for the proportion of endogenous DNA content. Endogenous content was calculated with SAMtools "*flagstat*" tool [36] and plotted as a function of collection year.

Fragment length

We quantified DNA fragmentation using two approaches. Firstly, to validate that the fragment sizes of the merged reads reflects the original molecule length, we examined the relationship between the fragment size distribution of isolated gDNA and that of the amplified libraries for a subset of the samples using TapeStation profiles for both (*N*= 40; 10 *H. spontaneum*, 10 *H. vulgare*, 10 *O. rufipogon*, and 10 *O. latifolia*; supplementary table S2). Library and gDNA peak size measurements from the TapeStation profiles were compared with each other and with bioinformatically-derived median fragment lengths of merged reads to assess the concordance between direct physical measurements and computational estimates. In addition, we further analysed the correlation between peak sizes of the gDNA isolates and age of the sample. Secondly, for the whole dataset (*N*= 573), we fitted the fragment length distribution of the mapped merged reads to a lognormal distribution using the 'fitdistr' function from the MASS package [40] in R. We used the mean of this distribution (log-mean) to summarise fragment

length for each library and carried out regressions on the relationship between the log-mean of fragment lengths and collection year. As suggested in [27], we used the median plotted on a log-scaled y-axis for visualisation, as the latter is more intuitive to understand than the log-mean.

Damage fraction per site (λ) and DNA decay rate (k)

The damage fraction per site (λ) was calculated for each sample using previously described methods [12]. We fitted an exponential decay model to the empirical fragment length distribution of the mapped reads and derived λ as the negative of the slope coefficient of the linear regression. To identify samples deviating from the assumption of exponential decay of fragment length, we assessed goodness of fit and statistical significance on a per-sample basis. Model fit was considered satisfactory when $R^2 > 0.95$ and $p < 0.05$.

Having determined the damage fraction per site (λ), we calculated genus-specific DNA decay rates (k) by plotting λ as a function of sample age and extrapolating k from the slope of the linear regression, according to the linear relationship $k = \lambda/age$ [41].

Nucleotide misincorporations

The frequency of 5' C>T substitutions at first position was used as a proxy for 5' damage. Although the depth of sequencing needed for reliable estimation of C>T frequencies is sample-dependent, an order of magnitude of thousands of reads is considered sufficient [42]. Therefore, only samples with >5,000 merged paired reads were retained for further analyses. Furthermore, the exponential increase of C>T substitutions at reads termini is routinely used as a metric to validate authenticity of aDNA [5]. We scored this pattern for each sample by fitting an exponential model to the C>T frequencies for the first 20 bases at 5' terminus. We evaluated

the goodness of fit with a one-sided *t*-test on a per-sample basis, as described in [43], and only retained samples that fitted the exponential model ($R^2 > 0.5$, $p < 0.05$).

Analysis of covariance

To investigate differences in aDNA damage patterns between *Hordeum* and *Oryza*, we performed Analysis of Covariance (ANCOVA) using the 'anova' function in R. For each aDNA damage metric, sample age was used as the covariate and the genus as the factor using the model "*y ~ covariate* x *factor*", which also tested for possible interactions between sample age and genus (i.e., differences in the slope of regression are dependent on genus). We tested this model against a model of type "*y ~ covariate + factor*" to assess whether the removal of the interaction influenced model fit. When the interaction was not significant ($p > 0.05$) we accepted the simpler model without interaction and concluded that regression slopes did not differ between genera, though intercepts might [27].

Climate analyses

We obtained climate data from the CHELSA V2.1 climate dataset [44] to investigate the relationship between specimen preservation conditions, aDNA damage patterns, and climatic variables. Four primary bioclimatic variables were extracted for each sample location based on latitude and longitude metadata: (i) annual mean temperature (bio1), (ii) temperature seasonality (bio4), (iii) annual precipitation (bio12), and (iv) precipitation seasonality (bio15). Additionally, we extracted monthly temperature (tas_01 - tas_12) and monthly precipitation (pr_01 - pr_12) data for each sample location to infer climatic conditions at the time of specimen collection. To quantify the unique and shared contributions of multiple explanatory variables to the total variance in aDNA damage metrics, we performed a variance partitioning analysis using the 'varpart' function implemented in the VEGAN package [45]. For each aDNA

metric, we applied a "collection climate" model, where monthly climatic variables (temperature and precipitation) were assigned to samples based on their location and month of collection, and an "annual climate" model, where annual temperature and precipitation means and their seasonality were assigned to samples based on their geographical location. Additionally, we included in this analysis the genus as a possible confounding effect and herbarium, which could be interpreted as different storage conditions. Since 'varpart' function accommodates only up to four variables, for some analysis, temperature and precipitation were merged into single climatic variable. For each response (endogenous DNA fraction, fragment size, lambda and 5' C>T damage), we tested statistical significance of individual fractions and combinations using redundancy analysis with the 'rda' function implemented in VEGAN, with 999 permutation followed by ANOVA.

Results:

A total of 573 herbarium specimens were sequenced, generating libraries sequenced to variable read counts and depth (supplementary table S1). *Hordeum* samples (*N* = 228) processed at the Royal Botanic Gardens, Kew (RBGK) generated an average of 12.08 ± 5.94 million reads per library, with 86.2 ± 24.3% of reads mapping to the relevant reference genome. *Oryza* samples (*N*= 58) processed at the RBGK yielded an average of 1.41 ± 0.93 million reads per library, with 86.5 ± 14.6% of reads mapping to the relevant refence genome. The remaining *Oryza* samples processed at the University of Nottingham (*N*= 287) generated an average of 0.06 ± 0.09 million reads per library, with 78.0 ± 26.2% of reads mapping to the relevant reference genome. Damage pattern analysis revealed characteristic aDNA signatures. *Hordeum* samples exhibited an average 5' C>T frequency of 1.09 ± 0.44% at the first base of sequenced molecule, whilst *Oryza* samples exhibited an average of 2.13 ± 0.70%. These damage signatures, combined with fragment length distributions, were used to authenticate samples and filter out

those inconsistent with genuine aDNA characteristics. Our filtering strategy identified samples deviating from the assumption of exponential decay of fragment length, as well as samples that did not display an exponential increase of C>T substitutions at read termini diagnostic of aDNA. In total, 117 (20%) samples were removed, leaving a final dataset containing 456 samples.

Regression analyses

Endogenous fraction

We analysed the proportion of reads mapping to the reference genome as a proxy for endogenous DNA content. The regression analyses revealed no statistically significant relationship between the proportion of endogenous DNA and the sample collection year in *Hordeum* (R^2 = 0.003, p = 0.451, N = 211), but a very weak yet significant relationship was observed in *Oryza* (R^2 = 0.04, p = 0.00167, N= 245; figure 2a). As we aimed at investigating the effect of genus on the rates of aDNA damage, we also carried the regression for all samples irrespective of genera, which provided comparable results (R^2 = 0.012, p = 0.0215, N= 456, supplementary figure S1a).

Fragment length

We measured DNA fragment size using two complementary approaches. First, we validated our bioinformatic estimates of fragment size distributions using TapeStation profiles of both gDNA and amplified libraries for a subset of samples (N= 40). Peak size of amplified libraries strongly correlated with the bioinformatically-derived median fragment size of the merged library reads (R^2 = 0.614, p =2.22 x 10^{-9}, supplementary figure S2a), compared to the weaker correlation between the TapeStation peaks of libraries and gDNA origin, (R^2 = 0.129, p =0.029, supplementary figure S2b). A significant, albeit weaker correlation was also observed between

the gDNA peak size and the median fragment size of merged reads (R^2 = 0.287, p =6.35 x 10^{-4}, supplementary figure S2c), indicating that merging of overlapping reads of short insert libraries reflects, at least in part, the original molecule length [29]. The weaker correlation was expected, as library preparation and sequencing involve processing and purification steps that can impact the fragment size distribution. Furthermore, we observed a strong and significant relationship between gDNA peak size and collection year (R^2 = 0.61, p =3.04 x 10^{-8}, N= 40, figure 3), but only after removing the two *Hordeum* outliers HV0061 and HV0081, which were particularly old (collection years: 1842 and 1867, respectively) but showed gDNA size distribution patterns inconsistent with their age, possibly due to modern contamination of exogenous DNA. With the inclusion of these two outliers a significant but weaker relationship (R^2 = 0.263, p =1.19 x 10^{-3}, supplementary figure S3) was observed.

Second, we extended this analysis to the full dataset and examined the relationship between bioinformatically-derived fragment size and age. We observed a statistically significant relationship between the log-mean fragment length and the sample collection year for both genera (figure 2b), with a stronger relationship for *Hordeum* (R^2 = 0.27, p =5.33 x 10^{-16}, N= 211) than *Oryza* (R^2 = 0.112, p = 8.58 x 10^{-8}, N= 245). A statistically significant relationship was also observed when analysing all samples irrespective of genera (R^2 = 0.171, p = 3.16 x 10^{-20}, N= 456; supplementary figure S1b).

Damage fraction per site (λ) and DNA decay rate (k)

The slope of log-transformed exponential decline of fragment length frequencies in aDNA (λ) describes the probability of bond breaking in DNA backbone [41]. We estimated the DNA decay rate per year (k) for *Hordeum* and *Oryza* from the slope of the linear relationship between λ and sample age (figure 2c). We observed a per nucleotide decay rate of k= 2.64 x 10^{-4} per year for *Hordeum* (R^2 = 0.208, p =3.27 x 10^{-12}, N= 211), which was 1.5 times faster than the

decay rate of *Oryza* of k= 1.79 x 10^{-4} per year (R^2 = 0.101, p = 3.65 x 10^{-7}, N= 245). The overall decay rate for all herbarium samples was k= 2.08 x 10^{-4} per year (R^2 = 0.129, p = 2.52 x 10^{-15}, N= 456, supplementary figure S1c), which is slightly faster than the decay rate of k= 1.66 x 10^{-4} per year observed in *Arabidopsis* and *Solanum* herbarium specimens [27], approximately 2.2 times slower than the k = 4.6 × 10^{-4} per year observed in dry-pinned arthropod museum specimens [8], and nearly eight times faster than the rate of k= 2.71 x 10^{-5} observed for ancient moa bones [12].

Nucleotide misincorporations

Both genera displayed statistically significant increases in the frequencies of 5’ C>T substitutions at first position correlating with the age of the sample (figure 2d), with *Oryza* starting from a higher baseline of damage when compared to *Hordeum* and displaying a stronger relationship (R^2 = 0.303, p = 8.62 x 10^{-21}, N= 245 for *Oryza*, and R^2 = 0.207, p =3.63 x 10^{-12}, N= 211 for *Hordeum,* respectively). A slightly weaker yet significant relationship between 5’C>T substitutions and sample age was also observed when analysing all samples together (R^2 = 0.106, p = 1.11 x 10^{-12}, N= 456; supplementary figure S1d).

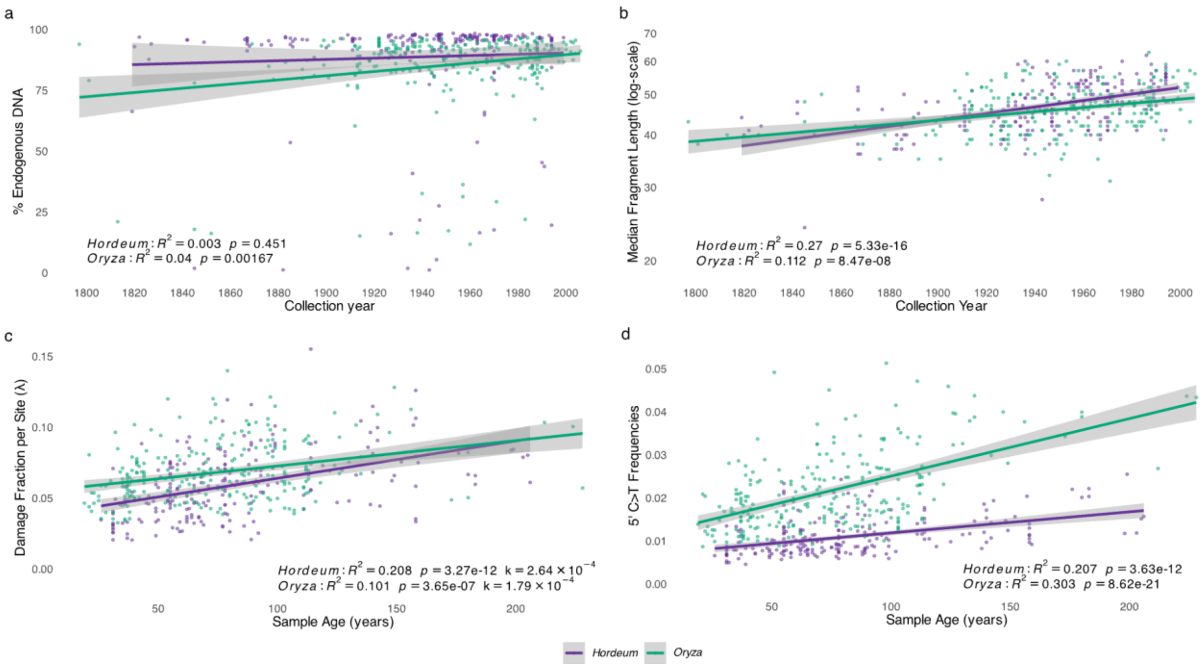

Figure 2: Regression analyses of aDNA damage metrics for *Hordeum* and *Oryza*: (a) Fraction of endogenous DNA as a function of collection year. (b) Median fragment length of merged reads as a function of collection year, with log-scaled y-axis to show exponential relationship. (c) Damage fraction per site (λ) as a function of sample age, with the slope of regression corresponding to the DNA decay rate per base per year for *Hordeum* ($k = 2.64 \times 10^{-4}$) and *Oryza* ($k = 1.79 \times 10^{-4}$). (d) Frequencies of C>T substitutions at first base (5'-end) as a function of sample age. Insets show regression statistics for each aDNA damage metric for each genus.

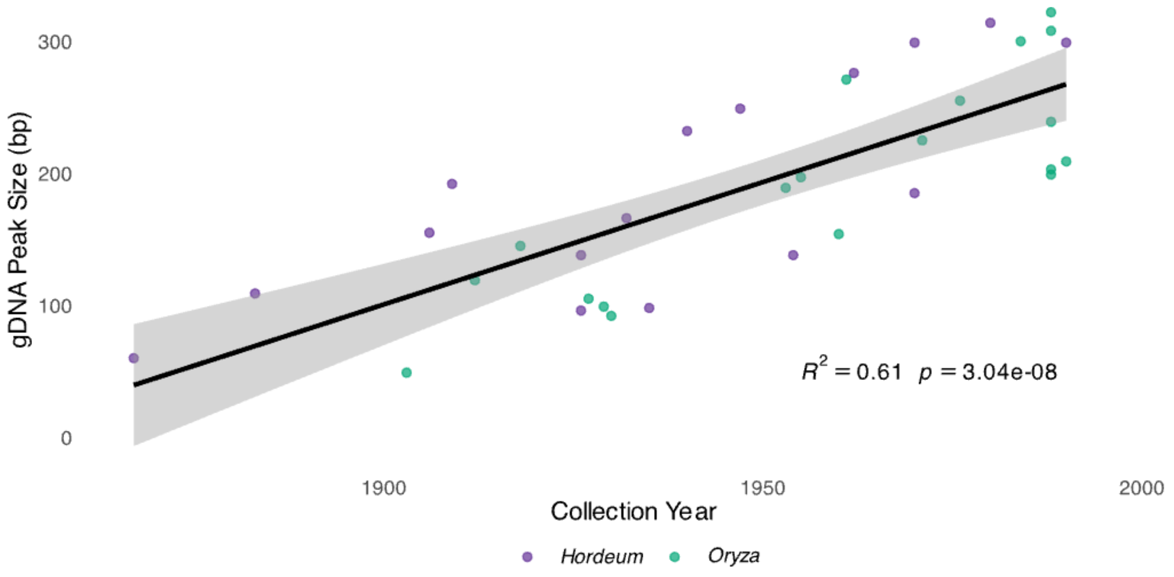

Figure 3: Regression between peaks of genomic DNA (gDNA) fragment size distribution obtained from high resolution fractionation electrophoresis (TapeStation) and collection year for a subset of samples (N = 40; 10 *H. spontaneum*, 10 *H. vulgare*, 10 *O. rufipogon*, and 10 *O. latifolia*;) after outlier removal. Inset shows regression statistics.

Differences in rates of damage for *Hordeum* and *Oryza*

We compared differences in the regression slopes and intercepts for all aDNA damage metrics between the genera *Hordeum* and *Oryza* with an analysis of covariance (ANCOVA) and visualised this comparison with boxplots.

Endogenous fraction

The analysis of covariance revealed significant effects of both sample age (*Pr*(Sample age) = 0.0114, N = 456) and genus (*Pr*(Genus) = 0.0204, N = 456) on the fraction of endogenous DNA (figure 4a). No significant interaction was observed between sample age and genus (*Pr*(Sample age: Genus) = 0.176, N = 456), indicating that the rate of exogenous DNA colonisation over time does not differ significantly between the two genera and is not time dependent. This was further supported by ANOVA between the model including the interaction and the model with no interaction, which confirmed that adding the interaction did not improve model fit (F = 1.841, Pr = 0.175). The model with no interaction displayed a significant fit (F= 5.395, Pr = 0.004835) but explained only a small proportion of the variance (R^2 = 0.02327), suggesting that factors beyond genus and sample age can substantially influence endogenous DNA content.

Fragment length

The analysis of covariance revealed significant effects of sample age and genus on fragment length (figure 4b) (Pr(Sample age) < 3.45×10^{-21}; Pr(Genus) = 0.0103; N = 456). The interaction between sample age and genus was not significant (Pr(Sample age: Genus) = 0.0893; N = 456) and its inclusion did not significantly affect model fit (F = 2.8993, Pr = 0.0904). The model with no interaction explained 18% of the variance in fragment length (R^2 = 0.1827) and displayed a significant fit (F = 50.65, $Pr < 2.2 \times 10^{-16}$), indicating that the genera differ in their baseline fragment lengths, but not in their rate of fragmentation over time.

Damage fraction per site (λ) and DNA decay rate (k)

We observed a slower DNA decay rate of *Oryza* (k= 1.79×10^{-4} per nucleotide per year) than that of *Hordeum* (k= 2.64×10^{-4} per nucleotide per year). The analysis of covariance revealed significant effects of both sample age (Pr(Sample age) = 7.48×10^{-18}; N = 456) and genus (Pr(Genus) = 5.18×10^{-8}; N = 456) on the rates of bond breaking (figure 4c). However, no interaction between sample age and genus was observed (Pr(Sample age: Genus) = 0.0884; N = 456) and including the interaction did not significantly improve model fit (F = 2.9152, Pr = 0.08843). The model with no interaction explained 18% of the variance in lambda values (R^2 = 0.1842) and displayed a highly significant fit (F = 51.14, $Pr < 2.2 \times 10^{-16}$). Therefore, the DNA decay rates (k) of *Hordeum* and *Oryza*, which correspond to the slopes of the regression between damage fraction per site (λ) and age, are not significantly different.

Nucleotide misincorporations

We observed a significant effect of both sample age (Pr(Sample age) = 3.66×10^{-6}; N = 456) and genus (Pr(Genus) = 3.14×10^{-4}; N = 456) on the rates of 5’ C>T substitutions (figure 4d). The interaction between sample age and genus was significant (Pr(Sample age: Genus) = 2.16×10^{-8}; N = 456), indicating that the rate of cytosine deamination over time differs substantially

between the two genera. The model with interaction explained almost 55% of the variance in 5'C>T damage ($R^2 = 0.5448$) and displayed a significant fit ($F = 180$, $Pr < 2.2 \times 10^{-16}$). This was further corroborated by the ANOVA analysis, which confirmed that including the interaction significantly improved model fit ($F = 32.49$, $Pr = 2.165 \times 10^{-8}$). Therefore, not only do *Hordeum* and *Oryza* samples differ in their baseline levels of cytosine deamination, but they also accumulate this type of damage at significantly different rates over time, with *Oryza* showing a steeper increase in deamination with age.

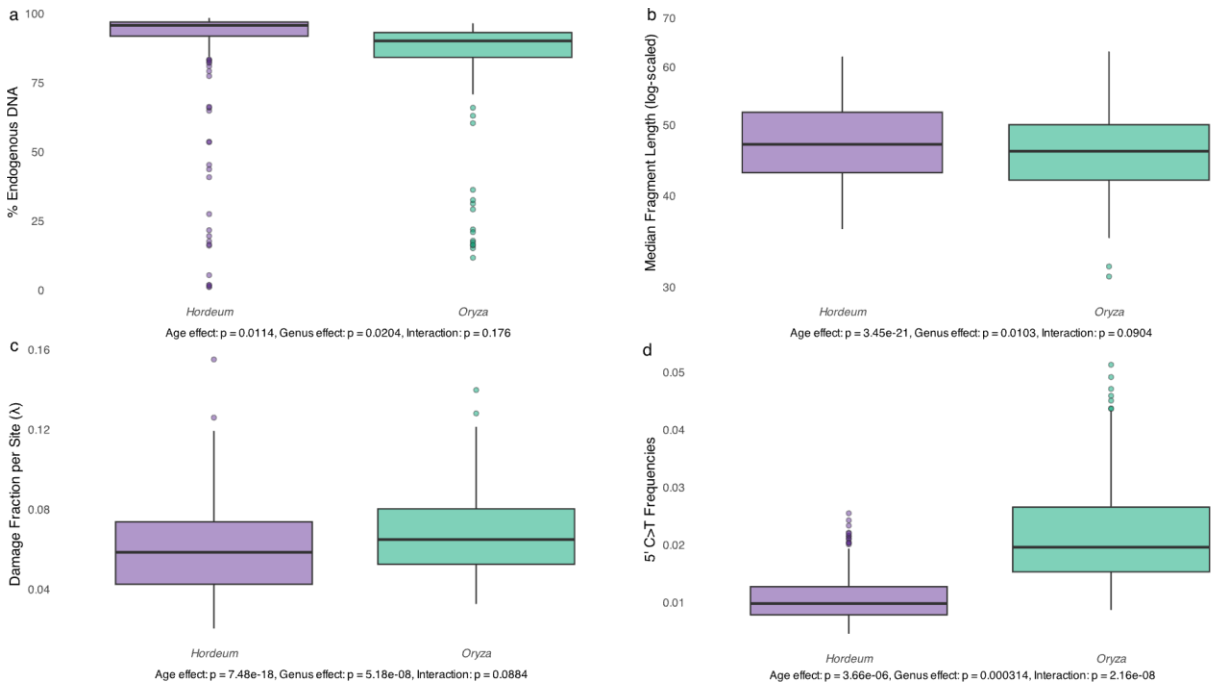

Figure 4: Analysis of Covariance (ANCOVA) of aDNA damage metrics for *Hordeum* and *Oryza*, with sample age as covariate and genus as factor. (a) Endogenous DNA fraction; (b) Fragment length (c) Damage fraction per site (λ); (d) 5' C>T damage frequencies. Significance values reported below each boxplot indicate the effects of age, genus and their interaction upon the analysed aDNA damage metric.

Effects of climatic variables upon rates of aDNA damage

We examined climatic effects using two complementary approaches: a "collection climate" model that assigned monthly temperature and precipitation values based on each sample's collection location and month, and an "annual climate" model that used annual means and seasonality measures based solely on geographical location. Variance partitioning analysis revealed distinct patterns of environmental and temporal control over different aspects of DNA preservation (figure 5). The relative importance of factors varied substantially among damage metrics, with some showing strong environmental sensitivity while others were primarily controlled by age-related processes. In addition to environmental factors, genus emerged as having significant explanatory power, possibly as a variable confounded with climate due to distinct distributions of the *Hordeum* and *Oryza* genera from temperate and tropical climates respectively (figure 1 and 5).

Endogenous fraction

Endogenous DNA content showed little association with the tested variables in the variance partitioning analysis (figure 5a). Only the collection climate model displayed statistical significance, but the fraction of variance explained by all analysed predictors was negligible (Adj. R^2 = 0.024, p = 0.007). The unique contributions of temperature and genus and the combined contributions of temperature, precipitation, age and genus showed marginal statistical significance ($p \leq 0.05$, supplementary table S3a). However, their poor performance in explaining the fraction of variance (Adj. R^2 ranging from 0.001 to 0.01) indicates that endogenous DNA fraction is largely determined by factors not captured by climatic variable nor age. Removing genus from the variance partitioning analysis did not significantly alter the results (supplementary figure S4a, supplementary table S4a). Similarly, inclusion of herbarium as a proxy of institutional preservation practices also did not significantly alter the results (supplementary figure S5a, supplementary table S5a).

Fragment length

Our models had more explanatory power when it came to fragment length, with both collection and annual climate factors being statistically significant ($p \leq 0.001$) and performing similarly (figure 5b, Adj. $R^2 = 0.227$ and 0.228, respectively). Sample age was revealed as the largest contributing factor in both collection and annual models (Adj. $R^2 = 0.199$, 0.193 respectively), consistent with temporal degradation processes. The shared variance between climatic variables and age were small, indicating that age effects on fragment size are largely independent of environmental variables. Indeed, whilst climatic variables were significant in both models (supplementary table S3b), their unique and combined variance explained were negligible (Adj. $R^2 \leq 0.01$). Removal of genus from the analysis marginally improved the unique and combined fraction of variance explained by temperature and precipitation in the collection and annual model respectively (supplementary figure S4b, supplementary table S4b), implying collinearity between genus and climatic variables, whereby further inclusion of herbarium had minimal effects (supplementary figure S5b, supplementary table S5b).

Damage fraction per site (λ)

Models predicting rates of DNA bond breaking (damage fraction per site, λ) from climatic and temporal variables explained up to 23% of the total variance in λ (figure 5c; Adj. $R^2 = 0.231$, 0.225 in the collection and annual models, respectively). Sample age contributed the largest unique fraction in both models (Adj. R^2 =: 0.170, 0.163), reflecting the fundamental relationship between specimen age and DNA backbone degradation. Temperature and precipitation showed minimal unique contributions and minimal shared variance with genus in the collection climate model (Adj. R^2 ranging from 0.01 to 0.03), and moderate shared variance with genus in the annual climate model (Adj. R^2 ranging from 0.03 to 0.04), suggesting indirect

effects through correlations between genus and climatic variables. Indeed, removal of genus from the analysis did not strongly affect the overall variance explained by each model but inflated the unique and shared variance explained by temperature and precipitation (supplementary figure S4c).

Nucleotide misincorporations

The 5' C>T damage metric showed the highest explained variance among all damage metrics, with both collection and annual climate models being significant ($p \leq 0.001$) and explaining approximately 49% of the total variance (figure 5d). Whilst the unique contribution of age emerged as the strongest predictor of 5' damage (Adj. $R^2 = 0.120$, 0.119 in the collection and annual models, respectively), most explanatory power derived from shared effects among predictors rather than unique contributions. The combined effects of temperature and genus explained a substantial proportion of the total variance (Adj. $R^2 = 0.135$, 0.097). A similar pattern was observed for the combined effect of precipitation and genus (Adj. $R^2 = 0.047$, 0.008) and for the combined effects of temperature, precipitation and genus (Adj. $R^2 = 0.096$, 0.213), suggesting a strong correlation between climatic variables and genus. Removal of genus from the analysis (supplementary figure S4d) revealed temperature as the largest unique contributing factor in the collection model (Adj. $R^2 = 0.134$), followed by sample age (Adj. $R^2 = 0.121$) and precipitation (Adj. $R^2 = 0.047$), whilst the combined effect of temperature and precipitation explained almost 10% of the variance ($R^2 = 0.096$). Notably, the variance explained by the combined effect of temperature and precipitation was inflated in the annual climate model (Adj. $R^2 = 0.214$, supplementary figure S4d) and emerged as the largest contributor, followed by unique contribution of sample age (Adj. $R^2 = 0.109$) and temperature (Adj. $R^2 = 0.096$). The inclusion of herbarium did not improve the total fraction of variance explained in neither model (supplementary figure S5d), with the unique and shared fraction of

explained variance by herbarium and other variables (age, climate and genus) being negligible (Adj. R^2 ranging from 0.0003 to 0.01).

Temperature effects on 5' C>T substitution frequencies

Given the predictive power of temperature in explaining 5' C>T damage patterns, we conducted post-hoc regression analyses to examine the relationships between temperature and cytosine deamination. We observed a strong positive relationship between temperature and 5' C>T substitutions (figure 6). The annual temperature model explained 18.4% of variance (R^2 = 0.184, p = 1.93 x 10^{-18}, N= 456, figure 6a), while the collection temperature model showed a slightly higher explanatory power (R^2 = 0.201, p = 3.74 x 10^{-20}, N= 456, figure 6b). However, this relationship was only observed when analysing the genera together and disappeared when analysing the genera on an individual basis (figure 6c, d).

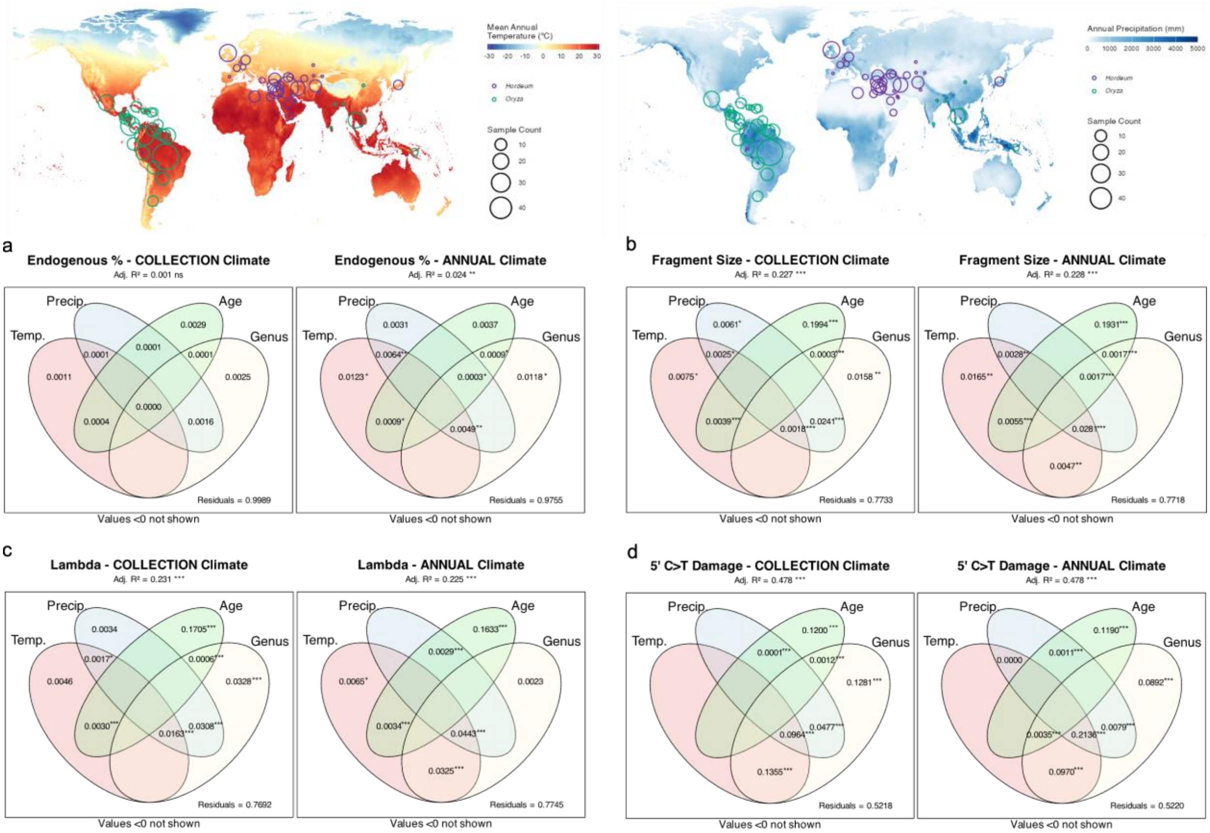

Figure 5: Climate influences on aDNA damage metrics in herbarium specimens. Maps (top panels) show the distribution of sampling locations for *Hordeum* and *Oryza* specimens overlaid on mean annual temperature (left) and annual precipitation (right) from the WorldClim climate dataset [46]. Venn diagrams (bottom panels) display the unique and shared contributions of the explanatory variables to the total variance in aDNA damage metrics.: (a) endogenous DNA fraction, (b) fragment size, (c) damage fraction per site (lambda), and (d) 5' C>T substitution frequencies at first base. Each metric is analysed using two models: collection climate (left) and annual climate (right). Adjusted R^2 values for each model are shown above the plots. Asterisks indicate statistical significance for the overall models and for each unique predictor and combination of predictors (*$p \leq 0.05$, **$p \leq 0.01$, ***$p \leq 0.001$).

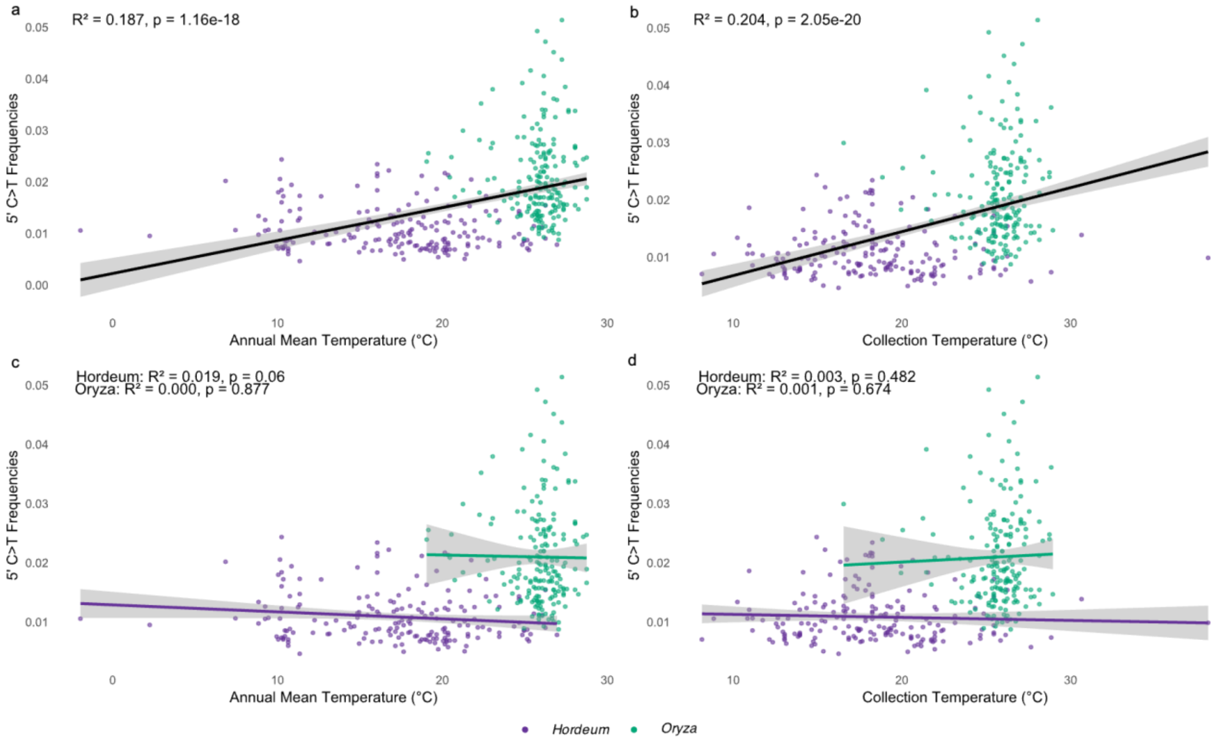

Figure 6: Relationship between temperature and deamination rates (5’ C>T substitution frequencies) in herbarium specimens for (a) annual mean temperature model, (b) collection temperature model, (c) annual mean temperature model for *Hordeum* and *Oryza*, and (d) collection temperature model for *Hordeum* and *Oryza*. Insets show regression statistics.

Discussion:

Genomic inferences from preserved samples allows scientists to directly add temporal scales to the studies of evolutionary and ecological histories of species, and of fundamental questions about the mechanisms and tempo of evolution. The advent of high-throughput sequencing technologies fuelled the revolutionary growth of the field of archaeo- and paleo-genomics. In 2022, the number of ancient human genomes passed 10,000 with over 200 papers published [19]. The discovery of extremally well-preserved samples [47] and development of new approaches [48–51] have pushed the limits of DNA recovery and sequencing. For plants, owing to the great diversity of wild and cultivated taxa, it is not possible to know the exact number of sequenced preserved specimens. In recent publication of 'Plant Tree of Life' alone, genome-wide enrichment sequences have been generated for over 2,500 plant genera from herbarium specimens [52]. Current projects focusing on crops and their wild relatives routinely sequence whole genomes for hundreds of historical specimens [53–55]. RGB Kew is currently aiming to sequence genomes of 7,000 preserved specimens of fungi from its fungarium collection [56]. With increasing scale of aDNA research across all kingdoms, it is fundamentally important to improve our understanding of DNA preservation in historical and archaeological samples.

The focus of aDNA preservation research has been the impact of time on DNA degradation. Thanks to two well-researched examples, we indeed know that DNA deamination and depurination is correlated with sample age [12,27]. However, it has been noted that environmental conditions pre- and post-mortem should modulate the pace at which DNA is degraded [2]. Emphasis has been put on temperature and humidity, two parameters that have been suggested as reasons for difficulties in sequencing ancient genomes from the tropics [2]. Our work on herbarium specimens has allowed us to quantify the impact of age, environment

and storage conditions on DNA preservation, revealing complex genus-specific differences extending beyond age-related degradation. Our regression analyses showed relationships between sample age and different damage metrics, suggesting that while some aspects of aDNA degradation follow predictable temporal trajectories, others are more heavily influenced by environmental and biological factors.

Age-dependent aDNA degradation patterns

We further confirmed the highly fragmented nature of aDNA retrieved from herbarium specimens, with the median fragment size of all analysed samples averaging 46.24 bp (SD = 6.97). This is consistent with previous studies on herbarium samples of a similar age range to that of the current study [27] and on dry-pinned arthropods museum collections [8], but also comparable to fragment sizes observed for animal remains that are several orders of magnitude older, from a few hundred up to thousands of years old [5,12]. Possibly owing to lower levels of environmental variation experienced by herbarium samples [27], we were able to detect a weak yet significant relationship between median fragment length and collection year (figure 2b). For a subset of samples ($N = 40$), we directly measured gDNA fragment size distributions prior to library preparation with high-resolution capillary electrophoresis (TapeStation), allowing us to assess DNA fragmentation independent of any library construction artifacts or bias. When examining the relationship between gDNA fragment size (TapeStation) and collection year, we found an even stronger correlation with age (figure 3). This contrasts with findings from animal bone studies, where no correlation between DNA fragmentation and sample age has been observed [5,12,57], even when controlling for environmental variables [2]. This fundamental difference can be explained by the two-step process of DNA decay and degradation: a first rapid phase of enzymatic decomposition occurring immediately after host death, driven by endogenous nucleases and proteases [58], and microbial digestion [59]. A

second phase of chemical decomposition driven by hydrolytic and oxidative reactions, occurring at much lower rates [6,7]. While the environment during sampling of herbarium specimens is variable, the standardized preparation and storage procedures used in herbaria reduce environmental variation during storage compared to the highly variable burial conditions experienced by animal bones. Indeed, the source herbaria (a proxy for storage conditions) have very limited explanatory power for DNA degradation (figure 5). The lower levels of environmental variation experienced by herbarium specimens allows detection of the underlying temporal fragmentation process that occurs during the second phase of chemical degradation [27,58]. In archaeological contexts, the influence of variable environmental, physical and chemical conditions, as well as tissue types and sample excavation and storage [2,12], may mask more subtle age-related fragmentation patterns that become detectable under the controlled conditions of herbarium storage.

Differences in physical and chemical tissue characteristics can explain the variation in DNA decay rates observed across different biological materials. Our herbarium samples exhibited decay rates ($k = 2.08 \times 10^{-4}$) nearly eight times faster than that observed in bones ($k = 2.71 \times 10^{-5}$;[12]), suggesting a higher susceptibility of *post-mortem* enzymatic and chemical DNA damage in herbarium samples compared to bones [27]. Interestingly, arthropod museum specimens show even faster decay ($k = 4.6 \times 10^{-4}$,[8]), approximately twice the rate observed in our herbarium samples. This variation likely reflects differences in tissue composition (cellulose/lignin in plants, chitin/protein in arthropods and hydroxyapatite/collagen in bones), storage methods (pressed herbarium sheets, pinned insects, and buried remains), and the protective properties of these different methods. The intermediate position of herbarium specimens between museum arthropods collections and ancient bones in terms of decay rate

suggests that DNA fragmentation is influenced by both intrinsic tissue properties and storage conditions.

Furthermore, the 5' C>T damage patterns also showed significant correlations with age in both genera (figure 2d). However, while both genera show similar rates of DNA fragmentation, their divergent responses to chemical modifications suggest different susceptibilities to oxidative damage and deamination. The higher baseline damage and steeper accumulation rates in *Oryza* species indicate that the genus is more susceptible to *post-mortem* deamination, possibly due them growing in different environments.

While aDNA damage metrics such as fragmentation and deamination were observed to be time-dependent processes, the fraction of endogenous DNA content showed no correlation with age (figure 2a), suggesting that microbial colonization and subsequent displacement of endogenous DNA is largely independent of specimen age. Biological degradation processes are thus primarily driven by factors other than time, such as initial microbial load, tissue characteristics, and individual storage conditions. However, whilst we used species-specific reference genomes, increasing evolutionary distance between query and reference might have impacted this metric [8]. We therefore suggest caution when interpreting such results.

Environmental controls on aDNA preservation

Our variance partitioning analysis shows that climatic effects on aDNA damage are largely mediated through complex interactions among predictors rather than independent contributions. The dominance of shared variance fractions indicates that temperature, precipitation and genus effects are highly correlated in our dataset, reflecting the geographic and temporal sampling patterns of herbarium collections.

For metrics reflecting DNA backbone integrity (fragment size and lambda), age consistently contributed the largest unique variance fractions, confirming that temporal degradation processes are the primary drivers of DNA fragmentation. The relatively large unique age contributions suggest that these physical degradation processes proceed independently of environmental and taxonomic factors once specimens enter standardized herbarium preparation methods storage practices. This age-dependent fragmentation is a distinctive feature of herbarium specimens that contrasts with the patterns observed in both archaeological animal bones [5,12] and arthropod museum collections [8], where environmental variation during deposition or storage can mask temporal patterns.

The strong correlation between temperature and 5' C>T damage provides mechanistic insight into why *Oryza* specimens, predominantly from tropical and sub-tropical regions, consistently show higher damage levels compared to temperate *Hordeum* specimens (figure 6). Elevated temperatures accelerate the spontaneous deamination of cytosine residues, explaining both the higher baseline damage observed in *Oryza* and the overall temperature-damage relationship observed across all herbarium samples. Precipitation variables as a proxy of humidity also showed significant effects, indicating that deamination is a time-dependent process modulated by temperature and humidity [2]. Overall, the complex interplay between temperature, humidity and their seasonality and damage metrics suggests that specimens from regions with less seasonal climates may experience different preservation trajectories than those from highly seasonal climates. The substantial unique genus contributions to 5' C>T damage confirm differential susceptibility to cytosine deamination between *Hordeum* and *Oryza*. However, the large shared variance fractions involving genus indicate that taxonomic differences are partially confounded with climatic variables, reflecting the contrasting geographic origins of these genera (temperate vs. tropical; figure 5). It is possible that genus captures other environmental

factors that were not included in temperature and precipitation. Precipitation is a decent proxy for humidity, but there are multiple other factors influencing it, and high humidity might be accelerating the deamination process. Alternatively, the differences observed due to tropical and temperate samples (captured in genus) could be explained by different sample processing approaches in the two areas, with tropical specimens being more often oven-dried/baked. Indeed, baking has been shown to substantially affect DNA degradation [60]. Additionally, in the tropics, alcohol treatment used to be common to prevent moulding, and this has been shown to limit the success in DNA amplification, presumably due to rapid degradation [61].

The negligible variance explained for endogenous fraction across all models supports our hypothesis that microbial colonization is largely independent of the measured environmental and biological predictors. This finding suggests that endogenous DNA loss is driven by stochastic factors such as initial contamination loads, handling procedures, or unmeasured specimen-specific characteristics.

Conclusions and implications for herbarium curation and ancient DNA research

We show that ancient DNA damage patterns result from complex interactions between temporal degradation processes, environmental conditions during sampling, biological properties and storage condition. Herbarium specimens exhibit age-dependent DNA fragmentation patterns that are not observed in animal bone studies, indicating that standardized preservation conditions can reveal underlying temporal degradation processes masked by environmental variation in archaeological contexts. We identified significant genus-specific differences in aDNA damage susceptibility and established temperature as the dominant environmental driver of cytosine deamination.

Environmental effects on DNA damage operate primarily through complex interactions with taxonomic and temporal factors rather than direct independent contributions. Age-related

degradation dominates physical DNA breakdown, while genus-specific differences in cytosine deamination susceptibility reflect both environmental adaptation and intrinsic biochemical properties. The comparison across biological materials reveals that tissue composition fundamentally constrains preservation potential, while storage conditions modulate decay rates.

These findings highlight the importance of considering specimen age, climatic origin and storage conditions when selecting herbarium specimens for ancient DNA analyses. The mechanisms underlying genus-specific differences in aDNA damage accumulation warrant detailed biochemical investigation. The significant, although small effect of herbarium-specific factors on damage metrics indicates that institutional preservation practices vary and represent an underexplored factor in ancient DNA research. Future studies should provide insights into the biological processes driving DNA degradation, including detailed microbiome analyses. Understanding which microbial taxa are most problematic for DNA preservation and how their colonization is influenced by specimen characteristics could inform targeted preservation strategies. As the scale of ancient DNA research continues to expand, such findings will be essential for maximizing the scientific value of the world's natural collections and informing evidence-based approaches to specimen sampling and preservation.

Availability of code and requirements:

Project name: Herbaria aDNA Damage

Project home page: https://github.com/Stefano-Porrelli/Herbaria_aDNA_Damage

Operating system(s): Linux/Unix (tested on SLURM-based HPC systems)

Programming language: Bash, R (version ≥4.0)

Other requirements:

- Conda/Miniconda,

- AdapterRemoval (≥2.0), BWA (≥0.7.17), FastQC (≥0.11), SAMtools (≥1.10), DeDup (≥0.12), mapDamage2 (≥2.0), MultiQC (≥1.8), Preseq (≥2.0), AMBER.
- R packages: dplyr, tidyr, purrr, stringr, readr, MASS, vegan, car, ggplot2, colorspace, viridis, ggrepel, ggtext, ggpubr, gridExtra, cowplot, sf, geodata, terra, maps
- External data: CHELSA V2.1 climate data (https://chelsa-climate.org/)

License: MIT licence

Any restrictions to use by non-academics: None

Data availability:

Raw FASTQ DNA sequences of all samples processed at the Royal Botanic Gardens, Kew (UK) are deposited on the sequence reads archive (SRA) on NCBI: *Hordeum vulgare* sequences are deposited under BioProject PRJNA1288534. *Hordeum spontaneum* sequences are deposited under BioProject PRJNA1289164. *Oryza rufipogon* sequences are deposited under BioProject PRJNA1288425. *Oryza grandiglumis* sequences are deposited under BioProject PRJNA1288424. *Oryza latifolia* sequences are deposited under BioProject PRJNA1288423. All remaining raw FASTQ DNA sequences of *Oryza* are deposited on the SRA under BioProject PRJNA1302186. Scripts and dataset to reproduce the analyses are available at https://github.com/Stefano-Porrelli/Herbaria_aDNA_Damage (EMBARGOED).

Supplementary material:

Supplementary material is available at XXXXXXX

Authors' contributions:

S.P. and R.M.G. conceived the project and designed the study; R.M.G. and S.P. designed the sampling strategy, with contributions from A.F. and P.H.L.; P.J.K and N.S. overseen and

supervised the generation of sequencing data for *Hordeum* samples; S.P. sampled herbaria and performed the aDNA laboratory work and bioinformatic screening for the *Hordeum* samples processed at RBGK; A.H. sequenced the *Hordeum* libraries at IPK; P.H.L. sampled herbarium samples and performed the aDNA laboratory work and bioinformatic screening for the *Oryza* samples processed at RBGK; A.F. selected herbarium specimens, A.F., M.N.R., and R.A.W. performed the sampling, A.F., W.Y., N.M., M.N.R., and A.C.C. isolated aDNA and prepared aDNA libraries, and A.F. performed screening and validation for the *Oryza* samples processed at KAUST/UoN; S.P. analysed the historical data and interpreted the results with the contribution of R.M.G. and P.H.L; S.P. and R.M.G. wrote the manuscript with contributions from all authors.

Acknowledgements:

We thank curators for providing herbarium specimens: Anna Haigh and Sue Zmartzy (Royal Botanic Gardens, Kew); Paul Peterson and Robert Soreng (Smithsonian Institute Herbarium); Jordan K. Teisher (Missouri Botanic Gardens); Miriam Gaudeul (National History Museum, France); Michaela Schmull and Anthony R. Brach (Harvard Herbarium); Matthew C. Pace (New York Botanic Gardens). We acknowledge the expert technical assistance of Ines Walde and Jacqueline Pohl during DNA sequencing of *Hordeum* samples at the Leibniz Institute of Plant Genetics and Crop Plant Research (IPK). We also thank Anne Fiebig for her expert technical assistance with data submission of *Hordeum* low-throughput SRA sequences at IPK.

Funding:

This work was supported by the European Union Horizon 2020 research and innovation programme under grant agreement No. 862613 (AGENT - Activated GEnebank NeTwork), which funded the processing and low-coverage sequencing of *Hordeum* samples at the Royal

Botanic Gardens, Kew and the Leibniz Institute of Plant Genetics and Crop Plant Research (IPK). UK Research and Innovation grant EP/X022404/1 funded the processing and low-coverage sequencing of *Oryza* samples at the Royal Botanic Gardens, Kew, while King Abdullah University of Science and Technology (KAUST) grant ORA-CRG10-2021-4734 to R.A.W. supported the processing and low-coverage sequencing of *Oryza* samples at the University of Nottingham.

Ethics and permissions:

Historical herbarium specimens were obtained from established herbaria following institutional specimen access protocols. All sampling was conducted under standard museum loan agreements and followed institutional guidelines for destructive sampling of herbarium material. No additional ethical permissions were required as the study involved only preserved specimens collected under historical botanical collecting practices prior to CBD, but authors adhere to the ethical principles for Access and Benefit Sharing.

Conflict of interest:

The authors declare no conflicts of interest.

References:

1. Kristiansen K. Towards a new paradigm? The third science revolution and its possible consequences in archaeology. *Curr Swed Archaeol*. Svenska Arkeologiska Samfundet; 2021; doi: 10.37718/csa.2014.01.
2. Kistler L, Ware R, Smith O, Collins M, Allaby RG. A new model for ancient DNA decay based on paleogenomic meta-analysis. *Nucleic Acids Res*. 2017; doi: 10.1093/nar/gkx361.

3. Orlando L, Allaby R, Skoglund P, Der Sarkissian C, Stockhammer PW, Ávila-Arcos MC, et al.. Ancient DNA analysis. *Nat Rev Methods Primers*. Springer Science and Business Media LLC; 2021; doi: 10.1038/s43586-020-00011-0.

4. Dabney J, Meyer M, Pääbo S. Ancient DNA damage. *Cold Spring Harb Perspect Biol*. Cold Spring Harbor Laboratory; 2013; doi: 10.1101/cshperspect.a012567.

5. Sawyer S, Krause J, Guschanski K, Savolainen V, Pääbo S. Temporal patterns of nucleotide misincorporations and DNA fragmentation in ancient DNA. *PLoS One*. Public Library of Science (PLoS); 2012; doi: 10.1371/journal.pone.0034131.

6. Lindahl T, Nyberg B. Rate of depurination of native deoxyribonucleic acid. *Biochemistry*. American Chemical Society (ACS); 1972; doi: 10.1021/bi00769a018.

7. Lindahl T. Instability and decay of the primary structure of DNA. *Nature*. Springer Science and Business Media LLC; 1993; doi: 10.1038/362709a0.

8. Mullin VE, Stephen W, Arce AN, Nash W, Raine C, Notton DG, et al.. First large-scale quantification study of DNA preservation in insects from natural history collections using genome-wide sequencing. *Methods Ecol Evol*. Wiley; 2023; doi: 10.1111/2041-210x.13945.

9. Lan T, Lindqvist C. Technical advances and challenges in genome-scale analysis of ancient DNA. *Population Genomics*. Cham: Springer International Publishing;

10. Schubert M, Ginolhac A, Lindgreen S, Thompson JF, Al-Rasheid KAS, Willerslev E, et al.. Improving ancient DNA read mapping against modern reference genomes. *BMC Genomics*. Springer Science and Business Media LLC; 2012; doi: 10.1186/1471-2164-13-178.

11. Dolenz S, van der Valk T, Jin C, Oppenheimer J, Sharif MB, Orlando L, et al.. Unravelling reference bias in ancient DNA datasets. *Bioinformatics*. Oxford University Press (OUP); 2024; doi: 10.1093/bioinformatics/btae436.

12. Allentoft ME, Collins M, Harker D, Haile J, Oskam CL, Hale ML, et al.. The half-life of DNA in bone: measuring decay kinetics in 158 dated fossils. *Proc Biol Sci*. The Royal Society; 2012; doi: 10.1098/rspb.2012.1745.

13. Hofreiter M, Paijmans JLA, Goodchild H, Speller CF, Barlow A, Fortes GG, et al.. The future of ancient DNA: Technical advances and conceptual shifts. *Bioessays*. Wiley; 2015; doi: 10.1002/bies.201400160.

14. Korneliussen TS, Albrechtsen A, Nielsen R. ANGSD: Analysis of next generation sequencing data. *BMC Bioinformatics*. Springer Nature; 2014; doi: 10.1186/s12859-014-0356-4.

15. Schubert M, Ermini L, Der Sarkissian C, Jónsson H, Ginolhac A, Schaefer R, et al.. Characterization of ancient and modern genomes by SNP detection and phylogenomic and metagenomic analysis using PALEOMIX. *Nat Protoc*. Springer Science and Business Media LLC; 2014; doi: 10.1038/nprot.2014.063.

16. Peltzer A, Jäger G, Herbig A, Seitz A, Kniep C, Krause J, et al.. EAGER: efficient ancient genome reconstruction. *Genome Biol*. Springer Science and Business Media LLC; 2016; doi: 10.1186/s13059-016-0918-z.

17. Willerslev E, Cooper A. Ancient DNA. *Proc Biol Sci*. The Royal Society; 2005; doi: 10.1098/rspb.2004.2813.

18. Liu Y, Bennett EA, Fu Q. Evolving ancient DNA techniques and the future of human history. *Cell*. Elsevier BV; 2022; doi: 10.1016/j.cell.2022.06.009.

19. Mallick S, Micco A, Mah M, Ringbauer H, Lazaridis I, Olalde I, et al.. The Allen Ancient DNA Resource (AADR) a curated compendium of ancient human genomes. *Sci Data*. Springer Science and Business Media LLC; 2024; doi: 10.1038/s41597-024-03031-7.

20. Frantz LAF, Bradley DG, Larson G, Orlando L. Animal domestication in the era of ancient genomics. *Nat Rev Genet*. Springer Science and Business Media LLC; 2020; doi: 10.1038/s41576-020-0225-0.

21. Peris D, Janssen K, Barthel HJ, Bierbaum G, Delclòs X, Peñalver E, et al.. DNA from resin-embedded organisms: Past, present and future. *PLoS One*. Public Library of Science (PLoS); 2020; doi: 10.1371/journal.pone.0239521.

22. Kistler L, Bieker VC, Martin MD, Pedersen MW, Ramos Madrigal J, Wales N. Ancient plant genomics in archaeology, herbaria, and the environment. *Annu Rev Plant Biol*. Annual Reviews; 2020; doi: 10.1146/annurev-arplant-081519-035837.

23. Estrada O, Breen J, Richards SM, Cooper A. Ancient plant DNA in the genomic era. *Nat Plants*. Springer Science and Business Media LLC; 2018; doi: 10.1038/s41477-018-0187-9.

24. : Index herbariorum - the William & Lynda Steere herbarium. The William & Lynda Steere Herbarium. http://sweetgum.nybg.org/ih/ (2018). Accessed 2025 Oct 23.

25. Burbano HA, Gutaker RM. Ancient DNA genomics and the renaissance of herbaria. *Science*. 2023; doi: 10.1126/science.adi1180.

26. Eckert L, Eckert I, Rahn O, So CP, Barrett RDH. Using herbarium collections to study genetic responses to global change. *New Phytol*. 2025; doi: 10.1111/nph.70454.

27. Weiß CL, Schuenemann VJ, Devos J, Shirsekar G, Reiter E, Gould BA, et al.. Temporal patterns of damage and decay kinetics of DNA retrieved from plant herbarium specimens. *R Soc Open Sci*. The Royal Society; 2016; doi: 10.1098/rsos.160239.

28. Latorre SM, Lang PLM, Burbano HA, Gutaker RM. Isolation, library preparation, and bioinformatic analysis of historical and ancient plant DNA. *Curr Protoc Plant Biol*. Wiley; 2020; doi: 10.1002/cppb.20121.

29. Kircher M, Sawyer S, Meyer M. Double indexing overcomes inaccuracies in multiplex sequencing on the Illumina platform. *Nucleic Acids Res*. Oxford University Press (OUP); 2012; doi: 10.1093/nar/gkr771.

30. Meyer M, Kircher M. Illumina sequencing library preparation for highly multiplexed target capture and sequencing. *Cold Spring Harb Protoc*. Cold Spring Harbor Laboratory; 2010; doi: 10.1101/pdb.prot5448.

31. Hofreiter M, Jaenicke V, Serre D, Haeseler A, Pääbo S. DNA sequences from multiple amplifications reveal artifacts induced by cytosine deamination in ancient DNA. *Nucleic Acids Res*. 2001; doi: 10.1093/NAR/29.23.4793.

32. Gutaker RM, Reiter E, Furtwängler A, Schuenemann VJ, Burbano HA. Extraction of ultrashort DNA molecules from herbarium specimens. *Biotechniques*. 2017; doi: 10.2144/000114517.

33. Briggs AW, Stenzel U, Meyer M, Krause J, Kircher M, Pääbo S. Removal of deaminated cytosines and detection of in vivo methylation in ancient DNA. *Nucleic Acids Res*. Oxford University Press (OUP); 2010; doi: 10.1093/nar/gkp1163.

34. Schubert M, Lindgreen S, Orlando L. AdapterRemoval v2: rapid adapter trimming, identification, and read merging. *BMC Res Notes*. Springer Nature; 2016; doi: 10.1186/s13104-016-1900-2.

35. Li H. Aligning sequence reads, clone sequences and assembly contigs with BWA-MEM. arXiv [q-bio.GN].

36. Danecek P, Bonfield JK, Liddle J, Marshall J, Ohan V, Pollard MO, et al.. Twelve years of SAMtools and BCFtools. *Gigascience*. Oxford University Press (OUP); 2021; doi: 10.1093/gigascience/giab008.

37. Jónsson H, Ginolhac A, Schubert M, Johnson PLF, Orlando L. mapDamage2.0: fast approximate Bayesian estimates of ancient DNA damage parameters. *Bioinformatics*. Oxford University Press (OUP); 2013; doi: 10.1093/bioinformatics/btt193.

38. Fornasiero A, Feng T, Al-Bader N, Alsantely A, Mussurova S, Hoang NV, et al.. Oryza genome evolution through a tetraploid lens. *Nat Genet*. Springer Science and Business Media LLC; 2025; doi: 10.1038/s41588-025-02183-5.

39. . R: language environment statistical computing. *R Foundation Statistical Computing*. Vienna, Austria;

40. Venables WN, Ripley BD, editors. Modern applied statistics with S-plus. New York, NY: Springer;

41. Deagle BE, Eveson JP, Jarman SN. Quantification of damage in DNA recovered from highly degraded samples--a case study on DNA in faeces. *Front Zool*. Springer Science and Business Media LLC; 2006; doi: 10.1186/1742-9994-3-11.

42. Gutaker RM, Burbano HA. Reinforcing plant evolutionary genomics using ancient DNA. *Curr Opin Plant Biol*. Elsevier BV; 2017; doi: 10.1016/j.pbi.2017.01.002.

43. Weiß CL, Dannemann M, Prüfer K, Burbano HA. Contesting the presence of wheat in the British Isles 8,000 years ago by assessing ancient DNA authenticity from low-coverage data. *Elife*. eLife Sciences Publications, Ltd; 2015; doi: 10.7554/eLife.10005.

44. Karger DN, Conrad O, Böhner J, Kawohl T, Kreft H, Soria-Auza RW, et al.. Climatologies at high resolution for the earth's land surface areas. *Sci Data*. Springer Science and Business Media LLC; 2017; doi: 10.1038/sdata.2017.122.

45. Oksanen J, Blanchet FG, Friendly M, Kindt R, Legendre P, McGlinn D, et al.. Vegan: community ecology package. *R package version 25-4*. 2019;

46. Fick SE, Hijmans RJ. WorldClim 2: new 1-km spatial resolution climate surfaces for global land areas. *Int J Climatol*. Wiley; 2017; doi: 10.1002/joc.5086.

47. van der Valk T, Pečnerová P, Díez-Del-Molino D, Bergström A, Oppenheimer J, Hartmann S, et al.. Million-year-old DNA sheds light on the genomic history of mammoths. *Nature*. Springer Science and Business Media LLC; 2021; doi: 10.1038/s41586-021-03224-9.

48. Dabney J, Knapp M, Glocke I, Gansauge M-T, Weihmann A, Nickel B, et al.. Complete mitochondrial genome sequence of a Middle Pleistocene cave bear reconstructed from ultrashort DNA fragments. *Proc Natl Acad Sci U S A*. Proceedings of the National Academy of Sciences; 2013; doi: 10.1073/pnas.1314445110.

49. Dabney J, Meyer M. Extraction of highly degraded DNA from ancient bones and teeth. *Methods in Molecular Biology*. New York, NY: Springer New York;

50. Gansauge M-T, Meyer M. Single-stranded DNA library preparation for the sequencing of ancient or damaged DNA. *Nat Protoc*. Springer Science and Business Media LLC; 2013; doi: 10.1038/nprot.2013.038.

51. Slon V, Hopfe C, Weiß CL, Mafessoni F, de la Rasilla M, Lalueza-Fox C, et al.. Neandertal and Denisovan DNA from Pleistocene sediments. *Science*. American Association for the Advancement of Science (AAAS); 2017; doi: 10.1126/science.aam9695.

52. Zuntini AR, Carruthers T, Maurin O, Bailey PC, Leempoel K, Brewer GE, et al.. Phylogenomics and the rise of the angiosperms. *Nature*. Springer Science and Business Media LLC; 2024; doi: 10.1038/s41586-024-07324-0.

53. Kistler L, de Oliveira Freitas F, Gutaker RM, Maezumi SY, Ramos-Madrigal J, Simon MF, et al.. Historic manioc genomes illuminate maintenance of diversity under long-lived clonal cultivation. *Science*. American Association for the Advancement of Science (AAAS); 2025; doi: 10.1126/science.adq0018.

54. Alsantely A, Gutaker R, Navarrete Rodríguez ME, Arrieta-Espinoza G, Fuchs EJ, Costa de Oliveira A, et al.. The International Oryza Map Alignment Project (IOMAP): the Americas-

past achievements and future directions. *J Exp Bot*. Oxford University Press (OUP); 2023; doi: 10.1093/jxb/erac490.

55. Kreiner JM, Latorre SM, Burbano HA, Stinchcombe JR, Otto SP, Weigel D, et al.. Rapid weed adaptation and range expansion in response to agriculture over the past two centuries. *Science*. American Association for the Advancement of Science (AAAS); 2022; doi: 10.1126/science.abo7293.

56. Varga T, Woods R, Pitsillides F, Hill R, Biketova AY, Llewellyn T, et al.. Whole genome sequencing of historical specimens from the world's largest fungal collection yields high-quality assemblies. *New Phytol*. Wiley; 2025; doi: 10.1111/nph.70472.

57. Pääbo S. Ancient DNA: extraction, characterization, molecular cloning, and enzymatic amplification. *Proc Natl Acad Sci U S A*. Proceedings of the National Academy of Sciences; 1989; doi: 10.1073/pnas.86.6.1939.

58. Molak M, Ho SYW. Evaluating the impact of post-mortem damage in ancient DNA: a theoretical approach. *J Mol Evol*. Springer Science and Business Media LLC; 2011; doi: 10.1007/s00239-011-9474-z.

59. Eglinton G, Logan GA. Molecular preservation. *Philos Trans R Soc Lond B Biol Sci*. The Royal Society; 1991; doi: 10.1098/rstb.1991.0081.

60. Staats M, Cuenca A, Richardson JE, Vrielink-van Ginkel R, Petersen G, Seberg O, et al.. DNA damage in plant herbarium tissue. *PLoS One*. Public Library of Science (PLoS); 2011; doi: 10.1371/journal.pone.0028448.

61. Särkinen T, Staats M, Richardson JE, Cowan RS, Bakker FT. How to open the treasure chest? Optimising DNA extraction from herbarium specimens. *PLoS One*. Public Library of Science (PLoS); 2012; doi: 10.1371/journal.pone.0043808.

Supplementary Figures

Click here to access/download
Supplementary Material
Supplementary_Figures_v3.docx

Supplementary Tables

Click here to access/download
Supplementary Material
Supplementary_Tables_v2.xlsx

Cover Letter
Click here to access/download;Personal Cover;cover-Letter_v1.0.docx

Royal Botanic Gardens Kew

Royal Botanic Gardens, Kew, Richmond TW9 3AE
020 8332 5000 | kew.org | info@kew.org

Xun Xu
Editor-in-Chief
BGI Research, Shenzhen, China
USA

Hongfang Zhang
Editor
GigaScience Press, BGI Shenzhen, China

21 October 2025

Dear Dr. Xu and Mrs. Zhang,

Following up on the positive response to our pre-submission enquiry, we would like to submit our manuscript titled "**Patterns of aDNA Damage Through Time end Environments – lessons from herbarium specimens**" for consideration as a research article in GigaScience Journal.

In our manuscript, we are investigating the dynamics of DNA mutation, decay and fragmentation in historical samples containing ancient DNA. Unlike other studies, especially those in archaeological research, we provide a large datasets with a global distribution, spanning 220 years, with unified and stable preservation condition and consistent sampling and laboratory processing procedures. We present new whole genome sequencing data for a total of 573 herbarium samples from 6 plant species, spanning the Americas and Eurasia, all processed with the exact same laboratory and bioinformatic protocol. We supplement the genomic data with geographic locations and environmental parameters (bioClim) which open the exploration of climatic factors that influence DNA decay. These datasets are expected to be of wide interest, allowing to mine for unknown degradation signatures in ancient and historical DNA and exploring the impact of anatomical features and environmental factors on DNA preservation. To highlight the utility of this dataset we have conducted analyses on the impact of sampling temperature on the DNA characteristic and show that it is the main contributor to DNA damage (spontaneous C-to-T conversions), substantially exceeding the contributions of sample age.

We declare no potential conflict of interest for all authors. Thank you for your consideration. We look forward to hearing favourably from you.

Yours sincerely,

Stefano Porrelli and Rafal Gutaker

Patron: His Majesty King Charles III
The Royal Botanic Gardens, Kew is an exempt charity.
