# Supplementary material for: Patterns of aDNA damage through time and environments—lessons from herbarium specimens
Source: Gigascience. 2026 Mar 5;15:giag026. doi: 10.1093/gigascience/giag026 (PMC13108254; doi:10.1093/gigascience/giag026)
Supplement: giag026_Authors_Response_To_Reviewer_Comments_original_submission [file giag026_authors_response_to_reviewer_comments_original_submission.pdf]

Author's Response To Reviewer Comments

Reviewer reports:

Reviewer #1: The manuscript by Stefano Porrelli and colleagues make a valuable contribution by scaling up previous work on
specimens and by exploring how collection environments influence patterns of aDNA degradation. The authors present a larg
specimens from six Hordeum and Oryza species spanning ~220 years and diverse climates. Using standardized ancient DNA p
resolution climate data, they model the effects of specimen age, collection environment, genus, and herbarium of origin on D
cytosine deamination.

The study robustly confirms that DNA fragmentation and λ are strongly age-dependent, that herbarium specimens exhibit de
arthropods, and that environmental factors (particularly temperature) appear to correlate with 5′ C→T damage when all sam
time, some aspects of the temperature interpretation, especially in relation to genus-level structure, merit further clarificatio
(herbarium identity) seem to have comparatively minor influence.

Overall I enjoyed reading this research, the dataset is rich, the methodological framework is strong, and the work has signific
understanding plant aDNA preservation in herbaria. I believe the paper merits publication, though several concerns should be
find bellow several points that I hope will help strengthen and refine the manuscript.

Author’s response: We thank the reviewer for their careful and thorough evaluation of our manuscript. We appreciate the rev
methodological framework, and core findings regarding age-dependent fragmentation, intermediate decay rates in herbarium
influence of storage conditions.
We particularly value the reviewer's constructive feedback regarding the interpretation of temperature effects in relation to g
correctly notes, the confounding between genus and temperature in our dataset (driven by the contrasting biogeographic orig
Hordeum) requires careful interpretation. We have substantially revised the Discussion to address this important point (see d
the global temperature-damage correlation is primarily driven by interactions with genus rather than temperature acting inde
We have carefully addressed all of the reviewer's concerns point-by-point below. We believe these changes have significantly
interpretability of our findings.

Major comments

Definition and calculation of endogenous DNA fraction

You define endogenous fraction as "the percentage of post-quality trimmed and merged reads for each sample mapped to its
say it "was calculated with SAMtools 'flagstat'" (line 206)
However, this is somewhat ambiguous:
Is the denominator the number of merged reads after AdapterRemoval, the total raw reads, or only non-duplicate mapped re
Author’s response: The denominator is the total number of adapter- and quality-trimmed merged reads (AdapterRemoval out
adapter- and quality-trimmed merged reads that mapped to each respective reference genome.
Do you include secondary/supplementary alignments (multi mappers), and how are PCR duplicates treated here?
Author’s response: BWA-aln reports only primary alignments by default (no secondary or supplementary alignments), so all r
decisions for each query sequence. This calculation was performed before PCR duplicate removal, meaning that PCR duplicate
estimates. This is a conscious decision to facilitate fair comparison, because raw/merged reads are also not deduplicated. We
metrics of DNA damage/decay (fragment length, decay rates (λ, k), and C>T substitution frequencies) are calculated from m
Given that endogenous fraction is one of your four key metrics (Methods, lines 197-200), it would be useful to make this com
We have clarified this methodological detail throughout the Methods section “Ancient DNA damage metrics and regression an
transparency about calculation of endogenous content and when deduplication was performed relative to different metrics ca
“The proportion of endogenous DNA was calculated for each sample as the fraction of quality- and adapter-trimmed merged r
reference genome divided by the total number of reads retained after adapter-trimming and quality filtering. Only primary ali
reads representing unique mapping decision for each query sequence. Since this metric can be impacted by increasing evolut
reference [9], we used the most closely related reference genome available for each of the species included in our dataset (ta
calculated with SAMtools “flagstat” tool [39] and plotted as a function of collection year.”

Need a better explanation of the "month of collection" variable

Lines 266-273: you state that monthly temperature and precipitation were extracted "to infer climatic conditions at the time
collection climate model variables were assigned "based on their location and month of collection."
Later, in the Results you again refer to "collection climate" and "annual climate" models (lines ~438-441).

However, it is not entirely clear whether month is explicitly included as a variable (e.g. as a categorical factor or via the corre
simply used the CHELSA monthly layer corresponding to the recorded month? Please clarify in the Methods how the month of
variable "month" per se, or is the only effect that you choose the relevant tas_XX and pr_XX layer?

This would make it much easier for readers to follow how "month" is used and what the collection climate actually represents
Author's response: We have now substantially revised both the Methods and Results sections to clarify how month of collectio
collection was not included as a separate categorical variable in the models. Rather, for each sample, we extracted temperatu
CHELSA monthly layers (tas_01 - tas_12 and pr_01 - pr_12) corresponding to the recorded month of collection at the sample
specimen collected in July would be assigned temperature and precipitation values from the tas_07 and pr_07 layers for its s
model thus represents the estimated climatic conditions at the time and place of specimen collection, while the "annual clima
conditions at each collection locality.
We have reworded the climate data extraction section for clarity (Methods section, (lines 312-316):
"Additionally, we retrieved monthly temperature (tas_01 - tas_12) and monthly precipitation (pr_01 - pr_12) means for year
to each sample's recorded month of collection and geographical location. We used this data to infer climatic conditions at the
referred as "collection climate"."
We have also expanded the variance partitioning analysis section (lines 317 -332) to explicitly explain the two complementar
" To quantify the unique and shared contributions of multiple explanatory variables to the total variance in aDNA damage me
analysis using the 'varpart' function implemented in the VEGAN package [50]. For each aDNA metric, we applied a "collection
variables (temperature and precipitation) were assigned to samples based on their recorded location and month of collection,
annual mean temperature, mean precipitation and their seasonality were assigned to samples based on their geographical lo
aimed at capturing DNA damage occurring during the initial post-collection period (field handling, drying, and early preservat
ambient environmental conditions, rather than during subsequent long-term storage under standardised herbarium condition
aimed at capturing the general climatic regime of the collection locality, thus providing a comparison to assess whether mont
explanatory power over annual averages. Annual climate variables may also better represent cumulative environmental expo
growing season climate) influence tissue properties relevant to DNA preservation."
We have added explicit framing in the results section to clearly define both models and make explicit that month serves as a
climate layer rather than a model variable itself. We clarified the conceptual distinction between our two modelling approache
" We examined climatic effects using two complementary approaches: a "collection climate" model that assigned monthly tem
on each sample's recorded collection location and month, and an "annual climate" model that used annual means and season
geographical origin. Both models aimed at capturing climate-driven DNA damage occurring during the initial post-collection p
preservation), rather than during subsequent long-term storage. This assumption is supported by two lines of evidence: first,
driven by endogenous nucleases and proteases and exogenous microbial digestion [51,52], which occurs immediately after h
mortem DNA damage [2,6,7,25]. Second, herbarium specimens are subsequently stored under standardised conditions that
[2,25]. We further tested this by including herbarium identity (as a proxy for institutional storage conditions) as a factor, and
for herbarium identity, supporting the premise that collection-time climate, rather than storage climate, is the relevant enviro
patterns. "

Need a clarification of "Collection Climate" vs. Herbarium Storage

In the Methods (lines ~271-274), you describe a collection climate model where "monthly climatic variables (temperature an
based on their location and month of collection," and an annual climate model based on annual means at the collection locati
this model relates to the actual time each specimen spent in the field vs. in herbarium storage. By definition, a 150-year-old
its lifetime in a collection, yet the climate used in the models is that of the collection locality at the time of sampling, not the
spent decades, despite the herbarium being included as a factor.

Could you please clarify explicitly what period of a specimen's "life after death" you intend to capture with the collection clima
post-mortem period, or are you also considering longer-term storage conditions in the herbarium?.
Author's response: The reviewer is correct that our climatic data focus on collection-time climate rather than long-term stora
explicit throughout the manuscript. This was previously briefly addressed in the Discussion section ("Environmental controls
that herbarium storage conditions are standardised and that the source herbarium has limited explanatory power, but we als
and results sections to clarify (see comment above).
Do you assume that most deamination and oxidative damage occur in the first days to months after collection, and that later

conditions contributes little to further degradation?
Author’s response: We did not assume that herbarium storage conditions have little effect on DNA damage, but our result are
herbarium has negligible effect on all parameters tested. We now discuss both in more details, as exemplified by lines 525-53
“We examined climatic effects using two complementary approaches: a "collection climate" model that assigned monthly tem
each sample's recorded collection location and month, and an "annual climate" model that used annual means and seasonalit
origin. Both models aimed at capturing climate-driven DNA damage occurring during the initial post-collection period (field ha
rather than during subsequent long-term storage. This assumption is supported by two lines of evidence: first, the rapid initia
endogenous nucleases and proteases and exogenous microbial digestion [51,52], which occurs immediately after host death,
damage [2,6,7,25]. Second, herbarium specimens are subsequently stored under standardised conditions that minimise furth
further tested this by including herbarium identity (as a proxy for institutional storage conditions) as a factor, and indeed fou
herbarium identity, supporting the premise that collection-time climate, rather than storage climate, is the relevant environm

Need for the integration of non-deamination mismatch controls and baseline divergence

Your analysis focuses on the aDNA-typical 5’ C→T misincorporations (Methods, lines 238-245; Results, lines 355-361).
However, you do not show any other mismatch frequencies (e.g. A→G, G→A etc) as a "negative control" to demonstrate that
decay, climate, age, genus effects) are specific to deamination rather than general elevation of error rates or mapping artefac
Author’s response: We have now included analyses of non-deamination substitution frequencies as negative controls to demo
cytosine deamination rather than general sequencing errors or mapping artifacts and revised the main manuscript throughou
S5, S6 and S9. We also refer to these in the main text (lines 432-437, 444-447, 624-627):
“Deamination frequencies at 5’ (C>T) and 3’(G>A) were highly correlated (R2 = 0.951, p = 7.14 x 10-161, N= 245 for Oryza
211 for Hordeum; supplementary figure S5a). This significant correlation remained largely unchanged even after correcting d
substitutions means (supplementary figure S5b). All samples that passed filtering showed the expected “mirrored” deaminati
(supplementary figure S5).”
“In stark contrast to the significant relationship between 5’ C>T misincorporation and age, non-deamination substitution rate
sample age (supplementary figure S6), indicating that the patterns observed are specific to post-mortem deamination rates.’
“Furthermore, whilst the relationship between non-deamination substitutions and temperature showed some significance, the
0.007 - 0.07, supplementary figure S9), indicating that the temperature-damage relationship we reported are specific to dea

On that specific point, Lines 622-624 and 651-653: You attribute the higher 5’ C>T frequencies in Oryza to greater susceptib
potentially linked to its tropical and sub-tropical distribution. However, because Oryza originates from consistently warmer re
temperate, genus and temperature are strongly confounded in your dataset. This is also supported by your own variance part
variance fractions (temperature × genus) indicate that these two predictors are difficult to disentangle.

Furthermore, Figure 6 shows that when analysing each genus separately, the relationship between either annual mean tempe
C>T frequencies is no longer significant. This suggests that the global temperature-damage correlation you report is largely d
than temperature acting independently or am I wrong ? Otherwise could you add a bit of discussion on that point to explain w
deamination, why do we not see this intra-genus with different temperature values?

The reviewer is correct that genus and temperature are strongly confounded in our dataset, as reflected in the substantial sha
partitioning analysis (Figure 6). We acknowledge that the global temperature-damage correlation is indeed driven primarily b
reviewer notes, Figure 7c,d shows that temperature relationships become non-significant when analysing each genus separat
the text and expanded the discussion to address this point (lines 799-818):

“The substantial unique genus contributions to 5' C>T damage confirm differential susceptibility to cytosine deamination betw
large shared variance fractions between genus and temperature reflect confounding effect of the contrasting geographic origi
figure 6). This geographic segregation limits our ability to fully disentangle genus-specific susceptibility from temperature-driv
examining temperature-damage relationships within each genus separately (figure 7 c,d), the correlations become non-signif
other environmental factors that were not included in temperature and precipitation. Precipitation is a decent proxy for humic
influencing it, and high humidity is expected to accelerate the deamination process. Alternatively, the differences observed du
(captured in genus) could be explained by different sample processing approaches in the two areas, with tropical specimens b
Indeed, baking has been shown to substantially affect DNA degradation [65]. Additionally, in the tropics, alcohol treatment us
and this has been shown to limit the success in DNA amplification, presumably due to rapid degradation [67]. Future studies
overlapping geographic distributions would be necessary to clearly separate genus-specific susceptibility from environmental
consistent genus-level difference we observe remains informative about the challenges of DNA preservation across different b

While I agree that environmental conditions at the time of collection may influence DNA degradation, another factor that coul
specific patterns is reference-read divergence. Indeed, in a recent unreviewed work (see preprint: https://doi.org/10.1101/20
percentage identity between the reference genome and the ancient reads can influence apparent damage estimates. Althoug
Hordeum/Oryza reads and their respective references is unlikely to be extreme given that plants do not evolve as rapidly as
the percentage average identity of each species per genus in the model) would help confirm that reference mismatch is not i
frequencies between genera.
Author's response: We appreciate the reviewer raising this consideration. We believe reference divergence is unlikely to be a
reasons:
(1) Species-specific reference alignment: All samples within each genus were aligned to species-appropriate, high-quality ref
any baseline reference mismatch within each genus. Critically, we used geographically appropriate references (O. glumipatula
Asian O. rufipogon reference for Asian samples) to further minimize reference bias. We now calculate divergence from the ref
substitutions that are not C-to-T or G-to-A (supplementary figure S1). We demonstrate that non-deamination substitution rat
effect on analysis by calculating divergence-corrected C-to-T rates (supplementary figure S6, S10, S11, S12).
(2) Minimal evolutionary timescale: Our specimens span only ~220 years of collection history. Plant genomes evolve orders o
microbial taxa examined in the cited preprint, making it implausible that meaningful evolutionary divergence has accumulate
Intraspecific nucleotide diversity in these crop wild relatives is typically far below the threshold that would generate systemat

---

Minor comments

Title : "Patterns of aDNA Damage Through Time end Environments" → "Time and Environments."
fixed

Line 95 - ex situ in italic.
fixed

Line 140 and elsewhere: Oryza should be in italics whenever used as a genus (same for Hordeum).
fixed

Line ~551: "extremally well-preserved samples" → "extremely well-preserved samples."
fixed

It may help to add one sentence acknowledging that classical laboratory negative controls (blank extractions) are not relevan
misincorporation spectra and MapDamage profiles effectively serve as authenticity checks (Methods, lines 176-187 and 238-
Author's response: We updated the Methods section to clarify the use of negative controls, their inclusion in laboratory work
contamination, their exclusion from regression analyses, and the role of MapDamage profiles as authenticity checks for the a
215):
"DNA extraction, library preparation and low throughput sequencing:
Laboratory steps for DNA extraction and library preparation for all 228 Hordeum (146 H. spontaneum and 82 H. vulgare) and
and 10 O. grandiglumis) (supplementary table S1) samples were carried out in a clean room facility at Royal Botanic Gardens
published best practices and aDNA protocol described in [31] with adjusted volumes. A further 287 Oryza samples (201 O. la
and 13 O. alta) (supplementary table S1) were processed in a clean room facility at the Ancient and Environmental DNA Labo
Nottingham (UoN, UK), following the same protocol and volume adjustments. Following best practices for aDNA research [3,3
alongside samples to monitor for environmental and reagent contamination. These included extraction blanks and library pre
"Negative laboratory controls (extraction and library blanks) were included in the bioinformatic screening to monitor/assess p
excluded from further analyses."

Discussion lines 641-648 compare herbarium specimens to bones and arthropods. It might help the reader if you add one exp
fragmentation relationships are detectable in herbaria but not in bones (standardized post-collection environment, as you nic
Author's response: we further reiterated this point as suggested by the reviewer. We have added clarifying sentences to the
directly address why age-fragmentation relationships differ between specimen types:
"For metrics reflecting DNA backbone integrity (fragment size and lambda), age consistently contributed the largest unique v
degradation processes are the primary drivers of DNA fragmentation. The relatively large unique age contributions suggest th
proceed independently of environmental and taxonomic factors once specimens enter standardized herbarium preparation me
dependent fragmentation appears to be a distinctive feature of museum specimens (such as herbarium specimens [25] and c
contrasts with the patterns observed in archaeological animal bones [5,13]. For the latter, variable burial or storage conditio

that can mask the underlying temporal degradation signal, whereas the standardised preparation and storage practices in mu
allowing age-related fragmentation patterns to emerge."

In Figure 6, consider adding a brief note in the legend stating that the strong relationship in panels a-b is largely driven by co
between genera, and that it disappears within genera (c-d). This would remind readers of the confounding you discuss in the
Author's response: as suggested, we have updated the figure legend (lines 642-649):
"Figure 7: Relationship between temperature and deamination rates (5' C>T substitution frequencies) in herbarium specimen
(b) collection temperature model, (c) annual mean temperature model for Hordeum and Oryza, and (d) collection temperatur
show regression statistics. The significant relationships observed when analysing all samples together (a, b) are largely driven
baseline damage levels between genera; these relationships are not significant when analysing each genus separately (c, d),
effects are confounded in this dataset."

In the Methods you state that you used linear models (lm) for regressions and varpart + rda for variance partitioning (lines 1
approach is reasonable, it would help to briefly address whether model assumptions (normality, homoscedasticity) were chec
transformed variables).
Author's response :Model assumptions were verified using standard diagnostic procedures (i.e. residual plots to assess norma
added this to the methods section (lines 225-227)
"Model assumptions were verified using standard diagnostic procedures. For all linear regressions, we examined residual plots
and homoscedasticity."

While the manuscript mentions storage effects in the discussion, it doesn't explore them in great detail. More focus on specifi
temperature, humidity control) might help contextualize the minor storage effects observed. A brief section or discussion on i
their variability could provide readers with more context about herbarium differences.
Author's response: Whilst we agree with the reviewer this might be an interesting avenue to investigate, unfortunately no rec
monitoring are available, they change in time with improved infrastructure, and institutional practices might differ between d
to rely on herbarium as the only proxy of summarized environmental conditions during storage.

Reviewer #2: Reproducibility report for: Patterns of aDNA Damage Through Time end Environments - lessons from herbariun
Journal: Gigascience
ID number/DOI: GIGA-D-25-00447
Reviewer(s): Laura Caquelin, Department of Clinical Neuroscience, Karolinska
Institutet, Sweden [Wrote the report and reproduced the results]
Gustav Nilsonne, Department of Clinical Neuroscience, Karolinska Institutet, Sweden
[Reviewed the final report]
----

1. Summary of the study
The authors evaluated DNA preservation in herbarium collections by analyzing genomic data from 573 specimens of Hordeum
degradation and identified factors affecting decay, finding that specimen age and environmental conditions strongly influence
----

2. Scope of reproducibility

According to our assessment the primary objective is: the regression analyses of aDNA damage metrics for Hordeum and Ory

- Outcome: "Four metrics were selected to quantify patterns of aDNA damage: (i) the proportion of endogenous DNA content
the damage fraction per site (λ), and (iv) the frequencies of 5' C>T substitutions." (lines 197-199)
- Analysis method outcome: "The four metrics were analysed in linear models as a function of collection year and sample age
200)

- Main result: The results of this outcome are presented in figure 2 "Regression analyses of aDNA damage metrics for Hordeu
302 to 361 in the "Regression analysis" section:
"Endogenous fraction
[…] The regression analyses revealed no statistically significant relationship between the proportion of endogenous DNA and t
= 0.003, p = 0.451, N = 211), but a very weak yet significant relationship was observed in Oryza (R2 = 0.04, p = 0.00167, N

Fragment length
[…] We observed a statistically significant relationship between the log-mean fragment length and the sample collection year
stronger relationship for Hordeum (R2 = 0.27, p =5.33 x 10-16, N=211) than Oryza (R2 = 0.112, p = 8.58 x 10-8, N= 245).

Damage fraction per site (λ) and DNA decay rate (k)
[…] We estimated the DNA decay rate per year (k) for Hordeum and Oryza from the slope of the linear relationship between
a per nucleotide decay rate of k= 2.64 x 10-4 per year for Hordeum (R2 = 0.208, p =3.27 x 10-12, N= 211), which was 1.5
of k= 1.79 x 10-4 per year (R2 = 0.101, p = 3.65 x 10-7, N= 245) […].

Nucleotide misincorporations
[…] (figure 2d), with Oryza starting from a higher baseline of damage when compared to Hordeum and displaying a stronger
21, N= 245 for Oryza, and R2 = 0.207, p =3.63x 10-12, N= 211 for Hordeum, respectively). […]"
----

3. Availability of Materials
a. Data
- Data availability: Raw data are not yet publicly available but uploaded in NCBI database. Processed data are shared via the
intermediate file is available on the GitHub repository.
- Data completeness: Complete processed data and intermediate file (all data necessary to reproduce main results are availa
- Access Method: Private journal dropbox and GitHub repository
- Repository: https://github.com/Stefano-Porrelli/Herbaria_aDNA_Damage
-Data quality: Structured
b. Code
- Code availability: Open
- Programming Language(s): R and Bash
- Repository link: https://github.com/Stefano-Porrelli/Herbaria_aDNA_Damage
- License: MIT license
- Repository status: Public
- Documentation: Clear Readme file. Additional details may be required to run the Bash code.

----

4. Computational environment of reproduction analysis

- Operating system for reproduction: MacOS 15.7.2
- Programming Language(s): R
- Code implementation approach: Using shared code
- Version environment for reproduction: R version 4.5.1/RStudio 2025.05.1

----

5. Results

5.1 Original study results
- Results 1: See screenshot figure 2:

5.2 Steps for reproduction

-> Run 01_Plant_aDNA_screening_prep.sh
- Issue 1: The reviewer link provided for the bioprojects on NCBI did not allow downloading.
-- Partial resolution: An email was sent to the authors requesting access to the raw data or sharing processed data and intern
via the private journal dropbox and intermediate file (aDNA_damage_screening_MAIN.txt) was shared both on the dropbox a

The authors contacted NCBI to enable downloading the raw data with the reviewer link, but no response has yet been receive
within a set timeframe, the computational reproducibility review was performed first using the processed data and then direc

Note: The two bash scripts were not run. Additional guidelines would be helpful for running these scripts, especially regarding

(changing the repository name or the link to the data for example).
Author’s response: We updated README.md on github repository with more detailed instructions on how to retrieve data and
Plant aDNA Pipeline (Latorre et. al., 2020)
The repository includes two bash scripts for processing raw sequencing data and compute damage metrics before running the
• 01_Plant_aDNA_screening_prep.sh: Sets up the environment and installs required software for running Plant_aDNA_pipelin
• 02_Plant_aDNA_screening_main.sh: Main pipeline to calculate aDNA damage metrics.
The screening pipeline assesses library quality, sequencing run quality, DNA degradation magnitude, endogenous DNA percen
quantification of nucleotide misincorporations
For a full description of the pipeline and laboratory methods, see the Plant_aDNA_pipeline Gitlab and associated publication (
Running the Plant aDNA Pipeline
The pipeline is designed to run on SLURM-based HPC systems. Before running, you'll need to configure a few variables and re
Step 1: Preparation Script (01_aDNA_screening_prep.sh)
This script creates the conda environment, sets up the directory structure, and downloads/indexes your reference genome.
Before running, edit lines 14-15:

```
PROJECT_NAME="ENTER_DIRECTORY_NAME" # e.g., "Hordeum_vulgare_RBGK"
REFERENCE_GENOME_FTP="FTP_LINK_TO_REF_GENOME" # e.g.,
"https://ftp.ncbi.nlm.nih.gov/genomes/all/GCA/949/783/245/GCA_949783245.1_Hvulgare_FT262_BPGv2/GCA_949783245.1
```

To run:

```
# Make it an executable:
chmod +x 01_aDNA_screening_prep.sh

# On SLURM systems:
sbatch 01_aDNA_screening_prep.sh

# On local systems (remove SBATCH headers first):
bash 01_aDNA_screening_prep.sh
```

Outputs: Directory structure with:
• 1_initial data/ - Initial raw data and indexed reference genome
• 2_trimmed_merged/ - Adapter-trimmed and merged reads
• 3_quality_control/ - FastQC reports
• 4_mapping/ - BAM files and mapping statistics
• 5_aDNA_characteristics/ - MapDamage2 outputs
• 6_AMBER/ - Mapping bias assessment
• 7_preseq/ - Complexity curves and yield predictions
Step 2: Main Pipeline (02_aDNA_screening_main.sh)
This script processes all samples through the aDNA screening workflow.
Before running, edit line 13:

```
PROJECT_NAME="ENTER_DIRECTORY_NAME" # Must match the name used in prep script, e.g. "Hordeum_vulgare_RBGK"
```

Prepare your input data:
• Place paired-end FASTQ files in the 1_initial_data/ directory
• Files must follow naming convention: [Sample]_1.fastq.gz and [Sample]_2.fastq.gz (Must match the names under "Sample
Retrieving Raw Sequencing Data
Raw FASTQ sequences are available from NCBI SRA. To download, use the SRA Toolkit (prefetch and fasterq-dump), for exan

```
# Install SRA Toolkit if needed
conda install -c bioconda sra-tools

# Download and convert to FASTQ (example for one BioProject)
prefetch PRJNA1288534
fasterq-dump PRJNA1288534 --split-files --outdir 1_initial_data/

# Compress the FASTQ files
gzip 1_initial_data/*.fastq
```

To run:

```
# Make it an executable:
chmod +x 02_aDNA_screening_main.sh

# On SLURM systems:
```

sbatch 02_aDNA_screening_main.sh

On local systems (remove SBATCH headers first):
bash 02_aDNA_screening_main.sh
Resource Requirements
The default SLURM settings request:
• Prep script: 16 CPUs, 32GB RAM, 24 hours
• Main script: 16 CPUs, 64GB RAM, 24 hours
Adjust these in the #SBATCH headers based on your system and dataset size.
Running Without SLURM
If running on a local system or non-SLURM HPC, remove or comment out lines 2-8 in each script (the #SBATCH directives) a

-> Run the analysis from the processed data shared
--> Run code aDNA_Dmg_Script00_collate_screening_results.r
- Issue 2: The code expects data organized in two sub-folders: 4_mapping and 5_aDNA_characteristics. Processed data were folders, each containing 4_mapping and 5_aDNA_characteristics.
-- Resolved: All data were merged manually into single 4_mapping and 5_aDNA_characteristics folders to match the script's to the readme file.
Author's response: We script aDNA_Dmg_Script00_collate_screening_results.r with a fuction to merge directories. updated R

- Issue 3: The sample_metadata.txt file was not correctly merged with the results dataframe. Many columns (Batch_no to X) contained NA values.
-- Resolved: A message was sent to the authors to resolve the issue. They updated both sample_metadata.txt and aDNA_da
Author's response: I have realised the problem stems from inconsistencies between sample naming conventions in the scree identifiers in the metadata file. Specifically, for the Hordeum samples, the directories are named using library IDs rather tha Oryza samples were missing their expected suffixes. This meant the left_join step failed to match metadata for those sample
I have now corrected this by updating the "Sample" column in the metadata file to reflect the actual directory names used in names are preserved in a "Sample_ID" column. I have uploaded the corrected sample_metadata.txt file to the GitHub reposi aDNA_damage_screening_MAIN.txt dataset on the GitHub repo to reflect these changes.
I have re-run the pipeline and it now works correctly. Please let me know if you encounter any further issues, and thank you

The reproduced aDNA_damage_screening_MAIN.txt file no longer contains NA values.

--> Run code aDNA_Dmg_Script02_Regressions.r: The script was run without any issues.

-> Run the analysis from the intermediate data file shared on Github
--> Run code aDNA_Dmg_Script02_Regressions.r: Run the code after renaming the file to aDNA_damage_screening_MAIN_s

5.3 Statistical comparison Original vs Reproduced results
- Reproduced results:
-- Using the processed data and the reproduced aDNA_damage_screening_MAIN.txt, the results of Figure 2 were successfully
-- Using the shared aDNA_damage_screening_MAIN.txt from GitHub, the results were also successfully reproduced (see scre

- Comments: Supplementary Figure 1 was also reproduced using the same code. We confirmed that the reproduced values m
Both the processed data and the intermediate data file reproduced Supplementary Figure 1 (see screenshots below).
- Errors detected: One reporting error was detected in the "Fragment length" section (line 336): the p-value for Oryza should reported in the text.
Author's response: updated line 336 with corrected p-value

- Statistical Consistency: All statistical results reproduced from both the processed data and the intermediate file are identica Comparison_reproduced_vs_original.csv and Comparison_two_reproductions.csv files attached with this report).

----

6. Conclusion
- Summary of the computational reproducibility review
The computational reproducibility review shows that the results in Figure 2 and related text of the original study were fully re

and the intermediate data file shared (aDNA_damage_screening_MAIN.txt). The statistical results reproduced are identical to
One minor reporting error was detected in the manuscript: the p-value for Oryza in the "Fragment length" section should be 8
Author's response: updated line 336 with corrected p-value

- Recommendations for authors
-- Provide clearer instructions for running the Bash scripts, including terminal commands and any manual steps.
Author's response: updated README.md on github (see above)
-- Ensure consistent sample naming across metadata files and data directories to avoid merging issues for all analysis/scripts
Author's response: corrected sample naming across metadata files (see above)
-- Consider making raw data publicly available or provide clear guidance for reviewers to access it.
Author's response: raw data submitted to SRA, embargoed until publication. As per SRA team response, reviewer link does no
metadata.
-- Maintain clear documentation of file structure to facilitate future reproducibility.
Author's response: updated README.md on github (see above)

Reviewer #3: I read this work with great interest, and I believe it represents an excellent contribution to our understanding o
for plants, since most studies in this field are usually carried out on animal tissues, bones, and similar materials. The authors
herbarium specimens results from a combination of temporal, environmental, and biological factors, with storage conditions a
that DNA fragmentation increases in dry plant tissue with sample age, it varies between genera, and that temperature is the
I agree with these interpretations, but the discussion can emphasize more the roles of water and oxidation in DNA degradatio
limits hydrolytic damage but may increase the oxidative processes, on the contrary, animal or arthropod specimens dry more
degradation dynamics. Considering these differences in the discussion can further clarify the mechanisms behind the observe
tissue types.

In the study, the methodologiies vare solid. The approaches used to estimate endogenous DNA content is appropriate, thoug
could strengthen the calculation. Methods for assessing DNA fragmentation, for DNA damage, and for decay rates, and 5' C→
for validating aDNA authenticity. The climate analyses also appear sound but I cannot provide detailed evaluations on this pa

The explanation for the correlation between fragment length and sample age it seems logical. Unlike animals, where DNA dec
death is instead gradual and diverse depending on tissue, and this allows enzymatic and microbial degradation to continue ov
strong age-fragmentation relationship.
Overall, the study highlights the importance of tissue type and storage conditions on DNA decay; however discussing how hy
between herbarium plants and other specimen types (animal) would further strengthen the interpretation of the decay rates.
Author's response: We thank Reviewer #3 for their enthusiastic assessment of our work and for recognizing its contribution t
plant materials. We are pleased that the reviewer found our methodologies solid and our interpretations well-supported by th

We particularly appreciate the reviewer's emphasis on the need to discuss hydrolytic versus oxidative degradation mechanism
revised the Discussion section to address this important mechanistic framework. We have added a new paragraph to the Disc
addresses:

- The two primary chemical mechanisms of DNA degradation (hydrolytic and oxidative).
- How rapid drying of herbarium specimens limits hydrolytic damage but may increase oxidative processes.
- The contrasting degradation dynamics in animal/arthropod specimens that dry more slowly.
- How these mechanistic differences help explain the decay rate variations we observe across specimen types.
-
"The distinct preservation pathways of herbarium specimens compared to bones and museum specimens also likely reflect dif
oxidative DNA degradation processes. Herbarium specimens are often dried with heat upon collection [43,65]. Whilst this rap
damage, it also increases rates of oxidative processes [6,43,65]. In contrast, animal bones or museum specimens such as pin
through gradual burial diagenesis or museum preparation. These mechanistic differences can contribute to the variation in DN
biological materials: our herbarium samples exhibited decay rates ($k = 2.08 \times 10^{-4}$) nearly eight times faster than that obser
suggesting a higher susceptibility of post-mortem enzymatic and chemical DNA damage in herbarium samples compared to b
museum specimens show even faster decay ($k = 4.6 \times 10^{-4}$,[9]), approximately twice the rate observed in our herbarium sa
differences in tissue composition (cellulose/lignin in plants, chitin/protein in arthropods and hydroxyapatite/collagen in bones
sheets, pinned insects, and buried remains), and the distinct degradation dynamics imposed by rapid versus gradual desiccat
herbarium specimens between museum arthropods collections and ancient bones in terms of decay rate suggests that DNA fr

tissue properties and storage conditions.”

This addition emphasizes that the distinct preservation pathways result from different balances between hydrolytic and oxidat shaped by drying rates during specimen preparation. We believe this mechanistic framework clarifies why herbarium specime between arthropod museum specimens and ancient bones, and strengthens the comparative aspect of our study.

We have also addressed all specific comments raised by the reviewer, including clarifications regarding terminology, sample p monitoring, and the rationale for analytical approaches.

Specific comments

The terminology related to ancient DNA preservation (e.g., DNA damage, DNA degradation, DNA decay) should be clarified ar text. These terms describe distinct processes, and specifying the intended meaning for each will improve precision and avoid refers to specific chemical lesions; DNA degradation describes the physical fragmentation of DNA molecules; and DNA decay which DNA deteriorates over time.

In view of the comment above on the terminology used, I believe that the sentence above conflates different processes: dear whereas depurination leads to DNA degradation through strand fragmentation. I suggest the terminology in the paper should Even if the authors do not wish to adopt this terminology I suggest that they clarify the terms more clearly at the beginning.
Author’s response: The reviewer is correct that clearer distinction between these terms would improve the manuscript. We ha following consistent terminology throughout:

DNA damage: Chemical modifications to nucleotides (e.g., cytosine deamination leading to C>T misincorporations). The term chemical modifications (primarily C>T deamination frequencies).

DNA degradation: Physical fragmentation of DNA molecules (e.g., backbone breaks from depurination and hydrolytic/oxidativ discussing physical fragmentation and fragment length.

DNA decay: The temporal process and rate at which DNA deteriorates over time (quantified by the decay rate constant k). th processes and decay rates (k, lambda).

We have corrected the sentence that conflated deamination and depurination (lines 62-68). The revised text now reads:

“The two most prominent post-mortem reactions associated with DNA damage and degradation are deamination and depurina absence of enzymatic repair machinery [6,7]. Deamination of cytosines into uracils leads to a characteristic pattern of ‘C>T’ r frequency at fragment termini [8], whilst depurination causes “nicks” (breakage) in the phosphodiester bonds and subsequer DNA fragmentation towards very small molecules (~30-100 bp).”

Line 106: …six plant species, spanning…
Author’s response: typo fixed

Line 98, 105: In this context, it is not appropriate to refer to deamination-induced substitutions as "mutations," since they re rather than random biological changes (mutations) that occurr in vivo. In addition, introducing this new term complicates eve previous comments.
Author’s response: We have removed all instances of "mutation" when referring to C>T misincorporations caused by post-mo manuscript, we now consistently use "DNA damage," "C>T misincorporations," "deamination," or "nucleotide misincorporation chemical modifications, reserving "mutation" exclusively for in vivo genetic changes where appropriate.

Line 116-118: I wonder if the sampling coverage for Hordeum, with highest counts in arid and warm regions, may be incomp Europe (e.g., Scandinavia) or Russia are not represented. These species are cultivated in Russia, Denmark, southern Sweden acknowledged as it could affect the generality of the conclusions especially regarding temperatures?

Author’s response: We agree with the reviewer that while we cover the majority of barley distribution, we did not cover it cor materials that are available at the collections we worked with. We now disclose this caveat in the materials and methods (Lin

“Although we aimed at representing the majority of environments for each species in our dataset, some parts of distribution r unavailability of samples in our collections.”

It is unclear why the study included only wild Oryza species (O. alta, O. grandiglumis, O. latifolia, O. rufipogon), whereas for
was used. Perhaps, including Oryza sativa can provide more information on DNA preservation in domesticated material and a
genera?

Author's response: The inclusion of both wild and cultivated Hordeum species but only wild Oryza species reflects several pra
samples were part of existing research projects focused on characterizing wild rice diversity and distribution patterns, which c
Also, herbarium availability varied between genera and our study design prioritized achieving broad species coverage, geogra
distribution over balancing domestication status between genera.
While we agree that including O. sativa would provide additional comparative data, our current design still allows meaningful
across genera. The domestication status of specimens is unlikely to significantly affect DNA degradation patterns, as operate
represents wild or cultivated forms. Our primary objective was to investigate how environmental factors and temporal factors
Importantly, we view this study as establishing a methodological and analytical framework that can be expanded in future wo
publicly available specifically to facilitate follow-up studies that incorporate additional genera, families, and specimen types pr
protocols. Such expanded datasets would allow systematic investigation of questions we could not address here, including do
multiple plant families, phylogenetic effects on DNA preservation, and tissue-type specific degradation patterns. The current s
data for these future comparative analyses across broader taxonomic and ecological gradients.

Table 1: Draw a line above the last row (Total)
fixed

Line 140: Oryza should be in italics
fixed

Line 140: why 58 Oryza (30 O. latifolia, 18 O. rufipogon and 10 O. grandiglumis)? Why not all Oryza samples.
Author's response: these 58 Oryza samples were processed in Clean Room facilities at RBGK (as for all the Hordeum samples
processed in Clean Room facilities at UoN. Whilst both projects were coordinated and the exact same protocol was used, we f
were processed (and sequenced) in different clean room facilities/sequencing facilities. We have reworded the methods for cla

From line 169, it appears that an additional 287 Oryza samples from different origins (KAUST) were used, but it is not clear (
specimens, and why this origin (KAUST) is not included in Table 1. Perhaps it would be better to explain at the beginning of t
samples and to clarify the content of Table 1 more clearly.
Author's response: All 573 samples are herbarium specimens collected from the seven herbaria listed in Table 1. However, th
DNA laboratory facilities:
228 Hordeum samples and 58 Oryza samples were processed at the Royal Botanic Gardens, Kew (RBGK) clean room facility.

287 Oryza samples were processed at the Ancient and Environmental DNA Laboratory (ÆDNA) at the University of Nottingha

Table 1 lists the herbaria from which herbarium tissue samples were collected (the specimen sources), not the laboratories w
processed or sequenced. KAUST is mentioned in the context of funding the processing and sequencing of the 287 Oryza samp
specimens itself. These specimens also originated from the herbaria listed in Table 1.

We have revised the Methods section to clarify the sample processing workflow and the distinction between herbarium source
We now explicitly describe the two processing batches (RBGK-processed vs. UoN-processed) and separated the description of
from laboratory processing facilities.

Line 143: it is not specified which part of the herbarium material was used. I assume leaves, but this should be clearly stated
Author's response: added "Leaf samples"

Line 149: Please clarify what "gDNA" refers to; genomic DNA? Since you spell out "genomic DNA" elsewhere in the paragraph
unnecessary.
Author's response: fixed

Line 149: Why was only a subset used? Please explain and provide a rationale.
Author's response: We used TapeStation analysis on a subset of gDNA isolates (N = 40) for two specific reasons:

1. Technical constraint: TapeStation D1000 analysis requires DNA concentrations of at least 5 ng/μL. Due to the degraded na
specimens, the majority of our samples had gDNA concentrations below this threshold (supplementary table 1). The 40 samp
were those with sufficient DNA concentration while still representing the full range of specimen ages and all four focal species
rufipogon, and 10 O. latifolia).

2. Methodological validation: A key objective was to validate that the bioinformatically-derived median fragment size from se
original gDNA fragment size distribution. This required direct comparison between pre-library gDNA fragment sizes (measure
fragment sizes (from bioinformatic analysis). The correlation we observed ($R^2 = 0.287$, $p = 6.35 \times 10^{-4}$; Supplementary Figu
estimates of fragment size are valid proxies for actual DNA fragmentation, justifying their use for the full dataset of 573 samp

While gDNA TapeStation analysis was limited to this subset, we did perform TapeStation D1000 analysis on all 573 amplified
to verify that library size profiles matched the expected target size (~180 bp) before sequencing.
This approach allowed us to validate our methodology while maximizing the number of samples included in the downstream a
estimates could be reliably obtained for all samples regardless of initial DNA concentration.

We have edited the methods to clarify.

Line 154: were the libraries constructed only on this subset as well?
Author's response: libraries were constructed for all 573 samples. We edited the main text for clarity (see further response b

Line 162: Fragment size: The first letter of the sentence should be capitalized.
Author's response: fixed

Lines 165-169: It is not clear for me how the different subsets of samples were used in this study. Here it is stated that all ba
were sequenced on NovaSeq in a specific place, whereas only 40 rice samples (from the initial subset of how many?) were se
at a different institute. Also, the 287 samples from KAUST are seqeunced on a MiSeq that has lower output compared to Nova
how the initial 573 samples were selected and used for all analyses.
Also, the 287 samples from KAUST were processed in an ancient DNA lab, but what about all the other samples? It would be
ancient DNA analyses was not used for all samples. In this regard, it should also be noted that the issue of contamination is
it was certainly considered by the authors; for example, by indicating whether negative controls (blank samples) were used a
the C>T signal ensures that we are dealing with authentic ancient sequences, but this should be highlighted and explained m
Author's response: We have substantially revised the Methods section to address these concerns:
Sample processing and sequencing : We have clarified the complete sample breakdown and processing details:

- All 573 samples were processed following the same standardized aDNA extraction and library preparation protocol [28]
- 228 Hordeum libraries (processed at RBGK) were sequenced on NovaSeq 6000 or NovaSeq X+ at IPK, Germany
- 58 Oryza libraries (processed at RBGK) were sequenced on Illumina NovaSeq X+ at Macrogen, Europe
- 287 Oryza samples were processed at the Ancient and Environmental DNA Laboratory (ÆDNA) at the University of Nottingh
UoN

We have also added this information for each sample in supplementary table 1.

We have removed ambiguous phrasing (e.g., "40 rice samples") that caused confusion about which samples were used for di
states "For all 573 samples" when describing library construction, and specifies when subsets were used for particular analys
TapeStation validation, which included representatives from all four focal species).

We acknowledge the reviewer's concern about specialized laboratory facilities. We want to clarify that all samples were proce
facility (either ÆDNA/UoN or RBGK, which both were design based on the same specifications within 2 years from one anothe
methods). All samples followed identical extraction and library preparation procedures.

We have added explicit text addressing contamination controls and authentication procedures (lines 160-162, 213-215):

"Following best practices for aDNA research [3,31], negative controls were processed alongside samples to monitor for enviro
These included extraction blanks and library preparation controls."

"Negative laboratory controls (extraction and library blanks) were included in the bioinformatic screening to monitor/assess p
from further analyses" (i.e. regressions)

We now explicitly state that MapDamage profiles served as authenticity checks, allowing identification and exclusion of sampl
signatures, in addition to exponential decay of fragment size. (Lines 274-283, lines 369-377)

We clarified that samples showing C>T damage patterns inconsistent with authentic aDNA were excluded from regression ana

Line 189: Why was it aligned to Oryza glumipatula (a new species not mentioned before?) and not against Oryza rufipogon?
Author's response: This alignment choice was made to minimize reference bias in our damage estimates, particularly for C>T
not recognized as a separate species but is taxonomically classified as South American O. rufipogon. However, South Americ
genetically distinct.

To avoid reference bias that could artificially inflate C>T damage estimates due to genuine nucleotide differences between qu
aligned each specimen to the most closely related available reference genome. Therefore, O. rufipogon samples collected fro
the O. glumipatula reference genome (PRJNA48429), which represents South American populations, while Asian O. rufipogon
rufipogon reference genome (PRJNA1029807). This approach ensures that observed C>T substitutions primarily reflect post-
evolutionary divergence between query and reference sequences.
We have clarified this in the Methods section and included O. glumipatula in Table 2 to document all reference genomes used

The authors report measuring gDNA fragment size distributions on a subset of 40 samples. It would be helpful if they could p
was chosen, and how it is representative of the full dataset, to clarify the rationale behind not analyzing all samples.
As addressed in our response to the earlier comment, the subset of 40 samples was selected based on both technical constra
objectives. The 40 sub-samples were chosen to be representative of the full dataset, including equal representation of all fou
range of collection years in the dataset and sufficient gDNA concentration (≥5 ng/μL) to meet TapeStation D1000 technical re

The primary purpose of this subset analysis was to validate that bioinformatically-derived fragment sizes from sequencing da
sizes. The strong correlation we observed between gDNA TapeStation measurements and bioinformatic estimates (R^2 = 0.28
S3c) confirmed that our bioinformatic approach provides valid estimates for the full dataset, justifying its use for all 573 sam

We have clarified this rationale in the Methods section as outlined in the response of the comments above to make explicit bo
and the validation purpose of this analysis.

Editorial Manager®

Cookie Preference Center

We use cookies which are necessary to make our site work. We may also use additional cookies to analyze, improve and personalize our content and your digital experience. For more information, see our Cookie Policy.

You may choose not to allow some types of cookies. However, blocking some types may impact your experience of our site and the services we are able to offer. See the different category headings below to find out more or change your settings.

Allow all

Manage Consent Preferences

Strictly Necessary Cookies

Always active

These cookies are necessary for the website to function and cannot be switched off in our systems. They are usually only set in response to actions made by you which amount to a request for services, such as setting your privacy preferences, logging in or filling in forms. You can set your browser to block or alert you about these cookies, but some parts of the site will not then work.

Functional Cookies

These cookies enable the website to provide enhanced functionality and personalisation. They may be set by us or by third party providers whose services we have added to our pages. If you do not allow these cookies then some or all of these services may not function properly.

Performance Cookies

These cookies allow us to count visits and traffic sources so we can measure and improve the performance of our site. They help us to know which pages are the most and least popular and see how visitors move around the site.

Targeting Cookies

These cookies may be set through our site by our advertising partners. They may be used by those companies to build a profile of your interests and show you relevant adverts on other sites. If you do not allow these cookies, you will experience less targeted advertising.

Cookie List

Clear

Apply Cancel

Consent Leg.Interest

Confirm my choices

Powe
