# Supplementary material for: Patterns of aDNA damage through time and environments—lessons from herbarium specimens
Source: Gigascience. 2026 Mar 5;15:giag026. doi: 10.1093/gigascience/giag026 (PMC13108254; doi:10.1093/gigascience/giag026)
Supplement: giag026_Reviewer_1_Report_original_submission — Reviewer 1 -- 11/25/2025 [file giag026_reviewer_1_report_original_submission.pdf]

Reviewer Report

Title: Patterns of aDNA Damage Through Time and Environments â€“ lessons from herbarium specimens

Version: Original Submission **Date:** 11/25/2025

Reviewer name: Reviewer 1

Reviewer Comments to Author:

The manuscript by Stefano Porrelli and colleagues make a valuable contribution by scaling up previous work on DNA damage in plant herbarium specimens and by exploring how collection environments influence patterns of aDNA degradation. The authors present a large-scale analysis of DNA damage in 573 specimens from six Hordeum and Oryza species spanning ~220 years and diverse climates. Using standardized ancient DNA protocols, shotgun sequencing, and high-resolution climate data, they model the effects of specimen age, collection environment, genus, and herbarium of origin on DNA fragmentation, decay rates, and cytosine deamination.
The study robustly confirms that DNA fragmentation and Î» are strongly age-dependent, that herbarium specimens exhibit decay rates intermediate between bones and arthropods, and that environmental factors (particularly temperature) appear to correlate with 5â€² Câ†’T damage when all samples are analysed together. At the same time, some aspects of the temperature interpretation, especially in relation to genus-level structure, merit further clarification (as detailed below). Storage conditions (herbarium identity) seem to have comparatively minor influence.
Overall I enjoyed reading this research, the dataset is rich, the methodological framework is strong, and the work has significant potential to become a reference for understanding plant aDNA preservation in herbaria. I believe the paper merits publication, though several concerns should be addressed prior to its acceptance. Please, find bellow several points that I hope will help strengthen and refine the manuscript.

Major comments
Definition and calculation of endogenous DNA fraction
You define endogenous fraction as "the percentage of post-quality trimmed and merged reads for each sample mapped to its respective reference" (lines 203-206) and say it "was calculated with SAMtools 'flagstat'" (line 206)
However, this is somewhat ambiguous:
Is the denominator the number of merged reads after AdapterRemoval, the total raw reads, or only non-duplicate mapped reads?
Do you include secondary/supplementary alignments (multi mappers), and how are PCR duplicates treated here?
Given that endogenous fraction is one of your four key metrics (Methods, lines 197-200), it would be useful to make this completely explicit.
Need a better explanation of the "month of collection" variable

Lines 266-273: you state that monthly temperature and precipitation were extracted "to infer climatic conditions at the time of specimen collection" and that in the collection climate model variables were assigned "based on their location and month of collection."
Later, in the Results you again refer to "collection climate" and "annual climate" models (lines ~438-441).
However, it is not entirely clear whether month is explicitly included as a variable (e.g. as a categorical factor or via the corresponding monthly raster) or whether you simply used the CHELSA monthly layer corresponding to the recorded month? Please clarify in the Methods how the month of collection enters the model. Is there a variable "month" per se, or is the only effect that you choose the relevant tas_XX and pr_XX layer?
This would make it much easier for readers to follow how "month" is used and what the collection climate actually represents.
Need a clarification of "Collection Climate" vs. Herbarium Storage
In the Methods (lines ~271-274), you describe a collection climate model where "monthly climatic variables (temperature and precipitation) were assigned to samples based on their location and month of collection," and an annual climate model based on annual means at the collection location. However, it is not clearly stated how this model relates to the actual time each specimen spent in the field vs. in herbarium storage. By definition, a 150-year-old specimen will have spent the majority of its lifetime in a collection, yet the climate used in the models is that of the collection locality at the time of sampling, not the climate of the herbarium building where it spent decades, despite the herbarium being included as a factor.
Could you please clarify explicitly what period of a specimen's "life after death" you intend to capture with the collection climate model? Is it mainly the drying/early post-mortem period, or are you also considering longer-term storage conditions in the herbarium?. Do you assume that most deamination and oxidative damage occur in the first days to months after collection, and that later storage in relatively stable herbarium conditions contributes little to further degradation?
Need for the integration of non-deamination mismatch controls and baseline divergence
Your analysis focuses on the aDNA-typical 5â€² Câ†’T misincorporations (Methods, lines 238-245; Results, lines 355-361).
However, you do not show any other mismatch frequencies (e.g. Aâ†’G, Gâ†’A etc) as a "negative control" to demonstrate that the patterns you report (exponential decay, climate, age, genus effects) are specific to deamination rather than general elevation of error rates or mapping artefacts.
On that specific point, Lines 622-624 and 651-653: You attribute the higher 5â€² C>T frequencies in Oryza to greater susceptibility to post-mortem deamination, potentially linked to its tropical and sub-tropical distribution. However, because Oryza originates from consistently warmer regions while Hordeum is predominantly temperate, genus and temperature are strongly confounded in your dataset. This is also supported by your own variance partitioning analysis, where large shared variance fractions (temperature Ã— genus) indicate that these two predictors are difficult to disentangle.
Furthermore, Figure 6 shows that when analysing each genus separately, the relationship between either annual mean temperature or collection temperature and 5â€² C>T frequencies is no longer significant. This suggests that the global temperature-damage correlation you report is largely driven by genus-level differences rather than temperature acting independently or am I wrong ? Otherwise could

you add a bit of discussion on that point to explain why if temperature does have an impact of deamination, why do we not see this intra-genus with different temperature values?
While I agree that environmental conditions at the time of collection may influence DNA degradation, another factor that could contribute to the observed genus-specific patterns is reference-read divergence. Indeed, in a recent unreviewed work (see preprint: https://doi.org/10.1101/2025.07.16.665190), showed that the percentage identity between the reference genome and the ancient reads can influence apparent damage estimates. Although divergence between the ancient Hordeum/Oryza reads and their respective references is unlikely to be extreme given that plants do not evolve as rapidly as microbial taxa, a sanity check (e.g., adding the percentage average identity of each species per genus in the model) would help confirm that reference mismatch is not inflating differences in estimated 5â€² C>T frequencies between genera.

Minor comments
Title : "Patterns of aDNA Damage Through Time end Environments" â†’ "Time and Environments."
Line 95 - ex situ in italic.
Line 140 and elsewhere: Oryza should be in italics whenever used as a genus (same for Hordeum).
Line ~551: "extremally well-preserved samples" â†’ "extremely well-preserved samples."
It may help to add one sentence acknowledging that classical laboratory negative controls (blank extractions) are not relevant to the regression models, but that misincorporation spectra and MapDamage profiles effectively serve as authenticity checks (Methods, lines 176-187 and 238-247).
Discussion lines 641-648 compare herbarium specimens to bones and arthropods. It might help the reader if you add one explicit sentence summarizing why age-fragmentation relationships are detectable in herbaria but not in bones (standardized post-collection environment, as you nicely explain in lines 595-603).
In Figure 6, consider adding a brief note in the legend stating that the strong relationship in panels a-b is largely driven by contrasting climates and baseline damage between genera, and that it disappears within genera (c-d). This would remind readers of the confounding you discuss in the text.
In the Methods you state that you used linear models (lm) for regressions and varpart + rda for variance partitioning (lines 197-201 and 269-281). While the overall approach is reasonable, it would help to briefly address whether model assumptions (normality, homoscedasticity) were checked for the linear regressions (e.g. on log-transformed variables).
While the manuscript mentions storage effects in the discussion, it doesn't explore them in great detail. More focus on specific herbarium storage methods (e.g., temperature, humidity control) might help contextualize the minor storage effects observed. A brief section or discussion on institutional preservation practices and their variability could provide readers with more context about herbarium differences.

Methods

Are the methods appropriate to the aims of the study, are they well described, and are necessary controls included? Choose an item.

Conclusions

Are the conclusions adequately supported by the data shown? Choose an item.

Reporting Standards

Does the manuscript adhere to the journal's guidelines on minimum standards of reporting? Choose an item.

Choose an item.

Statistics

Are you able to assess all statistics in the manuscript, including the appropriateness of statistical tests used? Choose an item.

Quality of Written English

Please indicate the quality of language in the manuscript: Choose an item.

Declaration of Competing Interests

Please complete a declaration of competing interests, considering the following questions:

- Have you in the past five years received reimbursements, fees, funding, or salary from an organisation that may in any way gain or lose financially from the publication of this manuscript, either now or in the future?
- Do you hold any stocks or shares in an organisation that may in any way gain or lose financially from the publication of this manuscript, either now or in the future?
- Do you hold or are you currently applying for any patents relating to the content of the manuscript?
- Have you received reimbursements, fees, funding, or salary from an organization that holds or has applied for patents relating to the content of the manuscript?
- Do you have any other financial competing interests?
- Do you have any non-financial competing interests in relation to this paper?

If you can answer no to all of the above, write 'I declare that I have no competing interests' below. If your reply is yes to any, please give details below.

I declare that I have no competing interests

I agree to the open peer review policy of the journal. I understand that my name will be included on my report to the authors and, if the manuscript is accepted for publication, my named report including any attachments I upload will be posted on the website along with the authors' responses. I agree for my

report to be made available under an Open Access Creative Commons CC-BY license (http://creativecommons.org/licenses/by/4.0/). I understand that any comments which I do not wish to be included in my named report can be included as confidential comments to the editors, which will not be published.

Choose an item.

To further support our reviewers, we have joined with Publons, where you can gain additional credit to further highlight your hard work (see: https://publons.com/journal/530/gigascience). On publication of this paper, your review will be automatically added to Publons, you can then choose whether or not to claim your Publons credit. I understand this statement.

Yes Choose an item.
