# Supplementary material for: Patterns of aDNA damage through time and environments—lessons from herbarium specimens
Source: Gigascience. 2026 Mar 5;15:giag026. doi: 10.1093/gigascience/giag026 (PMC13108254; doi:10.1093/gigascience/giag026)
Supplement: giag026_Reviewer_2_Report_original_submission — Reviewer 1 -- 11/26/2025 [file giag026_reviewer_2_report_original_submission.pdf]

Reviewer Report

Title: Patterns of aDNA Damage Through Time and Environments â€“ lessons from herbarium specimens

Version: Original Submission **Date:** 11/26/2025

Reviewer name: Reviewer 2

Reviewer Comments to Author:

Reproducibility report for: Patterns of aDNA Damage Through Time end Environments - lessons from herbarium specimens
Journal: Gigascience
ID number/DOI: GIGA-D-25-00447
Reviewer(s): Laura Caquelin, Department of Clinical Neuroscience, Karolinska Institutet, Sweden [Wrote the report and reproduced the results]
Gustav Nilsonne, Department of Clinical Neuroscience, Karolinska Institutet, Sweden [Reviewed the final report]
----
1. Summary of the study
The authors evaluated DNA preservation in herbarium collections by analyzing genomic data from 573 specimens of Hordeum and Oryza. They quantified DNA degradation and identified factors affecting decay, finding that specimen age and environmental conditions strongly influence DNA preservation.
----
2. Scope of reproducibility
According to our assessment the primary objective is: the regression analyses of aDNA damage metrics for Hordeum and Oryza.
- Outcome: "Four metrics were selected to quantify patterns of aDNA damage: (i) the proportion of endogenous DNA content, (ii) the fragment length distribution, (iii) the damage fraction per site (Î»), and (iv) the frequencies of 5' C>T substitutions." (lines 197-199)
- Analysis method outcome: "The four metrics were analysed in linear models as a function of collection year and sample age using the 'lm' function in R" (lines 199-200)
- Main result: The results of this outcome are presented in figure 2 "Regression analyses of aDNA damage metrics for Hordeum and Oryza" and in the related text lines 302 to 361 in the "Regression analysis" section:
"Endogenous fraction
[…] The regression analyses revealed no statistically significant relationship between the proportion of endogenous DNA and the sample collection year in Hordeum (R2 = 0.003, p = 0.451, N = 211), but a very weak yet significant relationship was observed in Oryza (R2 = 0.04, p = 0.00167, N= 245; figure 2a).
Fragment length
[…] We observed a statistically significant relationship between the log-mean fragment length and the sample collection year for both genera (figure 2b), with a stronger relationship for Hordeum (R2 = 0.27,

p =5.33 x 10-16, N=211) than Oryza (R2 = 0.112, p = 8.58 x 10-8, N= 245).
Damage fraction per site (Î») and DNA decay rate (k)
[…] We estimated the DNA decay rate per year (k) for Hordeum and Oryza from the slope of the linear relationship between Î» and sample age (figure 2c). We observed a per nucleotide decay rate of k= 2.64 x 10-4 per year for Hordeum (R2 = 0.208, p =3.27 x 10-12, N= 211), which was 1.5 times faster than the decay rate of Oryza of k= 1.79 x 10-4 per year (R2 = 0.101, p = 3.65 x 10-7, N= 245) […].
Nucleotide misincorporations
[…] (figure 2d), with Oryza starting from a higher baseline of damage when compared to Hordeum and displaying a stronger relationship (R2 = 0.303, p = 8.62 x 10-21, N= 245 for Oryza, and R2 = 0.207, p =3.63x 10-12, N= 211 for Hordeum, respectively). […]"

----

3. Availability of Materials
a. Data
- Data availability: Raw data are not yet publicly available but uploaded in NCBI database. Processed data are shared via the private journal dropbox, and the intermediate file is available on the GitHub repository.
- Data completeness: Complete processed data and intermediate file (all data necessary to reproduce main results are available).
- Access Method: Private journal dropbox and GitHub repository
- Repository: https://github.com/Stefano-Porrelli/Herbaria_aDNA_Damage
-Data quality: Structured
b. Code
- Code availability: Open
- Programming Language(s): R and Bash
- Repository link: https://github.com/Stefano-Porrelli/Herbaria_aDNA_Damage
- License: MIT license
- Repository status: Public
- Documentation: Clear Readme file. Additional details may be required to run the Bash code.

----

4. Computational environment of reproduction analysis
- Operating system for reproduction: MacOS 15.7.2
- Programming Language(s): R
- Code implementation approach: Using shared code
- Version environment for reproduction: R version 4.5.1/RStudio 2025.05.1

----

5. Results
5.1 Original study results
- Results 1: See screenshot figure 2:
5.2 Steps for reproduction
-> Run 01_Plant_aDNA_screening_prep.sh
- Issue 1: The reviewer link provided for the bioprojects on NCBI did not allow downloading.
-- Partial resolution: An email was sent to the authors requesting access to the raw data or sharing

processed data and intermediate files. Processed data were shared via the private journal dropbox and intermediate file (aDNA_damage_screening_MAIN.txt) was shared both on the dropbox and the GitHub repository.
The authors contacted NCBI to enable downloading the raw data with the reviewer link, but no response has yet been received. As the review needed to be performed within a set timeframe, the computational reproducibility review was performed first using the processed data and then directly with the intermediate file.
Note: The two bash scripts were not run. Additional guidelines would be helpful for running these scripts, especially regarding terminal commands and manual steps (changing the repository name or the link to the data for example).
-> Run the analysis from the processed data shared
--> Run code aDNA_Dmg_Script00_collate_screening_results.r
- Issue 2: The code expects data organized in two sub-folders: 4_mapping and 5_aDNA_characteristics. Processed data were received in several species-specific folders, each containing 4_mapping and 5_aDNA_characteristics.
-- Resolved: All data were merged manually into single 4_mapping and 5_aDNA_characteristics folders to match the script's requirements. This detail should be added to the readme file.
- Issue 3: The sample_metadata.txt file was not correctly merged with the results dataframe. Many columns (Batch_no to X) in aDNA_damage_screening_MAIN.txt contained NA values.
-- Resolved: A message was sent to the authors to resolve the issue. They updated both sample_metadata.txt and aDNA_damage_screening_MAIN.txt on GitHub.
Author's response:
"I have realised the problem stems from inconsistencies between sample naming conventions in the screening output directories and the sample identifiers in the metadata file. Specifically, for the Hordeum samples, the directories are named using library IDs rather than the short sample names, and some of the Oryza samples were missing their expected suffixes. This meant the left_join step failed to match metadata for those samples. Thank you for flagging this up.
I have now corrected this by updating the "Sample" column in the metadata file to reflect the actual directory names used in the screening outputs. The original short names are preserved in a "Sample_ID" column. I have uploaded the corrected sample_metadata.txt file to the GitHub repository, and also updated the aDNA_damage_screening_MAIN.txt dataset on the GitHub repo to reflect these changes.
I have re-run the pipeline and it now works correctly. Please let me know if you encounter any further issues, and thank you again for catching this."
The reproduced aDNA_damage_screening_MAIN.txt file no longer contains NA values.
--> Run code aDNA_Dmg_Script02_Regressions.r: The script was run without any issues.
-> Run the analysis from the intermediate data file shared on Github
--> Run code aDNA_Dmg_Script02_Regressions.r: Run the code after renaming the file to aDNA_damage_screening_MAIN_shared.txt.
5.3 Statistical comparison Original vs Reproduced results
- Reproduced results:
-- Using the processed data and the reproduced aDNA_damage_screening_MAIN.txt, the results of Figure 2 were successfully reproduced (see screenshots below).

-- Using the shared aDNA_damage_screening_MAIN.txt from GitHub, the results were also successfully reproduced (see screenshots below).
- Comments: Supplementary Figure 1 was also reproduced using the same code. We confirmed that the reproduced values match the original results.
Both the processed data and the intermediate data file reproduced Supplementary Figure 1 (see screenshots below).
- Errors detected: One reporting error was detected in the "Fragment length" section (line 336): the p-value for Oryza should be 8.47 x 10-8, not 8.58 x 10-8 as reported in the text.
- Statistical Consistency: All statistical results reproduced from both the processed data and the intermediate file are identical to those reported in the manuscript (see Comparison_reproduced_vs_original.csv and Comparison_two_reproductions.csv files attached with this report).
----
6. Conclusion
- Summary of the computational reproducibility review
The computational reproducibility review shows that the results in Figure 2 and related text of the original study were fully reproducible using both the processed data and the intermediate data file shared (aDNA_damage_screening_MAIN.txt). The statistical results reproduced are identical to those presented in the manuscript.
One minor reporting error was detected in the manuscript: the p-value for Oryza in the "Fragment length" section should be 8.47 Ã— 10â□»â□¸ instead of 8.58 Ã— 10â□»â□¸.
- Recommendations for authors
-- Provide clearer instructions for running the Bash scripts, including terminal commands and any manual steps.
-- Ensure consistent sample naming across metadata files and data directories to avoid merging issues for all analysis/scripts.
-- Consider making raw data publicly available or provide clear guidance for reviewers to access it.
-- Maintain clear documentation of file structure to facilitate future reproducibility.

Methods

Are the methods appropriate to the aims of the study, are they well described, and are necessary controls included? Choose an item.

Conclusions

Are the conclusions adequately supported by the data shown? Choose an item.

Reporting Standards

Does the manuscript adhere to the journal's guidelines on minimum standards of reporting? Choose an item.

Choose an item.

Statistics

Are you able to assess all statistics in the manuscript, including the appropriateness of statistical tests used? Choose an item.

Quality of Written English

Please indicate the quality of language in the manuscript: Choose an item.

Declaration of Competing Interests

Please complete a declaration of competing interests, considering the following questions:

- Have you in the past five years received reimbursements, fees, funding, or salary from an organisation that may in any way gain or lose financially from the publication of this manuscript, either now or in the future?
- Do you hold any stocks or shares in an organisation that may in any way gain or lose financially from the publication of this manuscript, either now or in the future?
- Do you hold or are you currently applying for any patents relating to the content of the manuscript?
- Have you received reimbursements, fees, funding, or salary from an organization that holds or has applied for patents relating to the content of the manuscript?
- Do you have any other financial competing interests?
- Do you have any non-financial competing interests in relation to this paper?

If you can answer no to all of the above, write 'I declare that I have no competing interests' below. If your reply is yes to any, please give details below.

1. no 2. no 3. no 4. no 5. no 6. no

I agree to the open peer review policy of the journal. I understand that my name will be included on my report to the authors and, if the manuscript is accepted for publication, my named report including any attachments I upload will be posted on the website along with the authors' responses. I agree for my report to be made available under an Open Access Creative Commons CC-BY license (http://creativecommons.org/licenses/by/4.0/). I understand that any comments which I do not wish to be included in my named report can be included as confidential comments to the editors, which will not be published.

Choose an item.

To further support our reviewers, we have joined with Publons, where you can gain additional credit to further highlight your hard work (see: https://publons.com/journal/530/gigascience). On publication of this paper, your review will be automatically added to Publons, you can then choose whether or not to claim your Publons credit. I understand this statement.

Yes Choose an item.
