# Supplementary material for: Patterns of aDNA damage through time and environments—lessons from herbarium specimens
Source: Gigascience. 2026 Mar 5;15:giag026. doi: 10.1093/gigascience/giag026 (PMC13108254; doi:10.1093/gigascience/giag026)
Supplement: giag026_Reviewer_3_Report_original_submission — Reviewer 3 -- 11/28/2025 [file giag026_reviewer_3_report_original_submission.pdf]

Reviewer Report

Title: Patterns of aDNA Damage Through Time and Environments â€“ lessons from herbarium specimens

Version: Original Submission **Date:** 11/28/2025

Reviewer name: Reviewer 3

Reviewer Comments to Author:

I read this work with great interest, and I believe it represents an excellent contribution to our understanding of aDNA preservation, particularly welcome for plants, since most studies in this field are usually carried out on animal tissues, bones, and similar materials. The authors show that ancient DNA (aDNA) damage in herbarium specimens results from a combination of temporal, environmental, and biological factors, with storage conditions affecting decay rates. Their results indicate that DNA fragmentation increases in dry plant tissue with sample age, it varies between genera, and that temperature is the main driver of cytosine deamination.
I agree with these interpretations, but the discussion can emphasize more the roles of water and oxidation in DNA degradation. Rapid drying of herbarium specimens limits hydrolytic damage but may increase the oxidative processes, on the contrary, animal or arthropod specimens dry more slowly, andthis allows different degradation dynamics. Considering these differences in the discussion can further clarify the mechanisms behind the observed patterns, especially across museum tissue types.
In the study, the methodologiies vare solid. The approaches used to estimate endogenous DNA content is appropriate, though applying a mapping quality threshold could strengthen the calculation. Methods for assessing DNA fragmentation, for DNA damage, and for decay rates, and 5' Câ†’T substitutions seem robust and oprimal for validating aDNA authenticity. The climate analyses also appear sound but I cannot provide detailed evaluations on this part due to limited expertise in this area.
The explanation for the correlation between fragment length and sample age it seems logical. Unlike animals, where DNA decay occurs in two phases, plant tissue death is instead gradual and diverse depending on tissue, and this allows enzymatic and microbial degradation to continue over longer periods, contributing to the strong age-fragmentation relationship.
Overall, the study highlights the importance of tissue type and storage conditions on DNA decay; however discussing how hydrolytic and oxidative processes differ between herbarium plants and other specimen types (animal) would further strengthen the interpretation of the decay rates.
Specific comments
The terminology related to ancient DNA preservation (e.g., DNA damage, DNA degradation, DNA decay) should be clarified and used more consistently throughout the text. These terms describe distinct processes, and specifying the intended meaning for each will improve precision and avoid confusion for the reader. DNA damage refers to specific chemical lesions; DNA degradation describes the physical fragmentation of DNA molecules; and DNA decay refers to the temporal process or rate at which DNA deteriorates over time.
The two most prominent reactions associated with DNA degradation are deamination (resulting with

spontaneous substitutions of cytosine residues to uracil) and depurination (breakage of the phosphodiester bond resulting in DNA backbone fragmentation).
In view of the comment above on the terminology used, I believe that the sentence above conflates different processes: deamination is a form of DNA damage, whereas depurination leads to DNA degradation through strand fragmentation. I suggest the terminology in the paper should be modified to reflect this distinction. Even if the authors do not wish to adopt this terminology I suggest that they clarify the terms more clearly at the beginning.
Line 106: …six plant species, spanning…
Line 98, 105: In this context, it is not appropriate to refer to deamination-induced substitutions as "mutations," since they represent post-mortem chemical damage rather than random biological changes (mutations) that occurr in vivo. In addition, introducing this new term complicates even more the terminology presented in the previous comments.
Line 116-118: I wonder if the sampling coverage for Hordeum, with highest counts in arid and warm regions, may be incomplete, as certain regions, such as northern Europe (e.g., Scandinavia) or Russia are not represented. These species are cultivated in Russia, Denmark, southern Sweden, I believe. Should this limitation be acknowledged as it could affect the generality of the conclusions especially regarding temperatures?
It is unclear why the study included only wild Oryza species (O. alta, O. grandiglumis, O. latifolia, O. rufipogon), whereas for Hordeum the cultivated Hordeum vulgare was used. Perhaps, including Oryza sativa can provide more information on DNA preservation in domesticated material and allow a more consistent comparison across genera?
Table 1: Draw a line above the last row (Total)
Line 140: Oryza should be in italics
Line 140: why 58 Oryza (30 O. latifolia, 18 O. rufipogon and 10 O. grandiglumis)? Why not all Oryza samples.
From line 169, it appears that an additional 287 Oryza samples from different origins (KAUST) were used, but it is not clear (not explained) if these are herbarium specimens, and why this origin (KAUST) is not included in Table 1. Perhaps it would be better to explain at the beginning of this paragraph that there are two subsets of samples and to clarify the content of Table 1 more clearly.
Line 143: it is not specified which part of the herbarium material was used. I assume leaves, but this should be clearly stated
Line 149: Please clarify what "gDNA" refers to; genomic DNA? Since you spell out "genomic DNA" elsewhere in the paragraph, the abbreviation here seems unnecessary.
Line 149: Why was only a subset used? Please explain and provide a rationale.
Line 154: were the libraries constructed only on this subset as well?
Line 162: Fragment size: The first letter of the sentence should be capitalized.
Lines 165-169: It is not clear for me how the different subsets of samples were used in this study. Here it is stated that all barley samples (but how many exactly?) were sequenced on NovaSeq in a specific place, whereas only 40 rice samples (from the initial subset of how many?) were sequenced on another NovaSeq platform and at a different institute. Also, the 287 samples from KAUST are seqeunced on a MiSeq that has lower output compared to NovaSeq. Somehow, it is necessary to explain how the initial 573 samples were selected and used for all analyses.

Also, the 287 samples from KAUST were processed in an ancient DNA lab, but what about all the other samples? It would be strange if a specialized laboratory for ancient DNA analyses was not used for all samples. In this regard, it should also be noted that the issue of contamination is not mentioned in the manuscript, although it was certainly considered by the authors; for example, by indicating whether negative controls (blank samples) were used and how they were processed. Certainly, the C>T signal ensures that we are dealing with authentic ancient sequences, but this should be highlighted and explained more clearly.
Line 189: Why was it aligned to Oryza glumipatula (a new species not mentioned before?) and not against Oryza rufipogon?
The authors report measuring gDNA fragment size distributions on a subset of 40 samples. It would be helpful if they could provide a motivation for why this subset was chosen, and how it is representative of the full dataset, to clarify the rationale behind not analyzing all samples.

Methods

Are the methods appropriate to the aims of the study, are they well described, and are necessary controls included? Choose an item.

Conclusions

Are the conclusions adequately supported by the data shown? Choose an item.

Reporting Standards

Does the manuscript adhere to the journal's guidelines on [minimum standards of reporting?] Choose an item.

Choose an item.

Statistics

Are you able to assess all statistics in the manuscript, including the appropriateness of statistical tests used? Choose an item.

Quality of Written English

Please indicate the quality of language in the manuscript: Choose an item.

Declaration of Competing Interests

Please complete a declaration of competing interests, considering the following questions:

- Have you in the past five years received reimbursements, fees, funding, or salary from an organisation that may in any way gain or lose financially from the publication of this manuscript, either now or in the future?
- Do you hold any stocks or shares in an organisation that may in any way gain or lose financially from the publication of this manuscript, either now or in the future?
- Do you hold or are you currently applying for any patents relating to the content of the manuscript?
- Have you received reimbursements, fees, funding, or salary from an organization that holds or has applied for patents relating to the content of the manuscript?
- Do you have any other financial competing interests?
- Do you have any non-financial competing interests in relation to this paper?

If you can answer no to all of the above, write 'I declare that I have no competing interests' below. If your reply is yes to any, please give details below.

I declare that I have no competing interests

I agree to the open peer review policy of the journal. I understand that my name will be included on my report to the authors and, if the manuscript is accepted for publication, my named report including any attachments I upload will be posted on the website along with the authors' responses. I agree for my report to be made available under an Open Access Creative Commons CC-BY license (http://creativecommons.org/licenses/by/4.0/). I understand that any comments which I do not wish to be included in my named report can be included as confidential comments to the editors, which will not be published.

Choose an item.

To further support our reviewers, we have joined with Publons, where you can gain additional credit to further highlight your hard work (see: https://publons.com/journal/530/gigascience). On publication of this paper, your review will be automatically added to Publons, you can then choose whether or not to claim your Publons credit. I understand this statement.

Yes Choose an item.
